# Misexpression of genes lacking CpG islands drives degenerative changes during aging

**DOI:** 10.1126/sciadv.abj9111

**Published:** 2021-12-15

**Authors:** Jun-Yeong Lee, Ian Davis, Elliot H. H. Youth, Jonghwan Kim, Gary Churchill, James Godwin, Ron Korstanje, Samuel Beck

**Affiliations:** 1 Davis Center for Regenerative Biology and Medicine, MDI Biological Laboratory, Bar Harbor, ME 04609, USA.; 2 Brown University, Providence, RI 02912, USA.; 3 Department of Molecular Biosciences, The University of Texas at Austin, Austin, TX 78712, USA.; 4 The Jackson Laboratory, Bar Harbor, ME 04609, USA.

## Abstract

Cellular aging is characterized by disruption of the nuclear lamina and its associated heterochromatin. How these structural changes within the nucleus contribute to age-related degeneration of the organism is unclear. Genes lacking CpG islands (CGI^−^ genes) generally associate with heterochromatin when they are inactive. Here, we show that the expression of these genes is globally activated in aged cells and tissues. This CGI^−^ gene misexpression is a common feature of normal and pathological aging in mice and humans. We report evidence that CGI^−^ gene up-regulation is directly responsible for age-related physiological deterioration, notably for increased secretion of inflammatory mediators.

## Teaser

Disrupted nuclear architecture triggers misexpression of genes lacking CpG islands and physiological deterioration during aging.

## INTRODUCTION

**Fig. 1.**
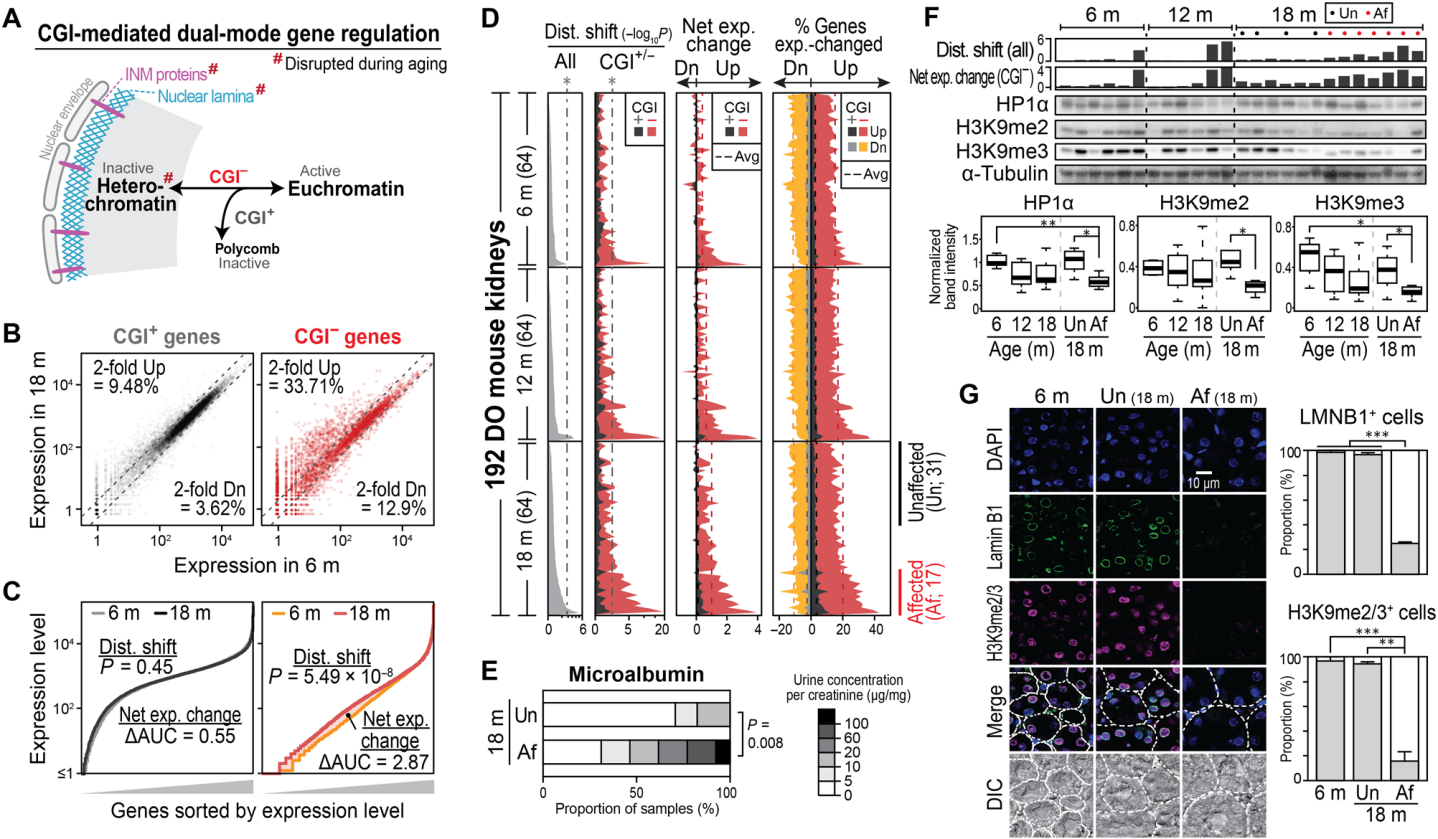
CGI^−^ gene misexpression and nuclear architecture disruption in aged kidneys. (**A**) Schematic showing dual mode gene regulation mediated by CGI. Only CGI^−^ genes form lamina-associated heterochromatin, which is disrupted during aging (see also fig. S1A). (**B**) Example comparison of CGI^+^ and CGI^−^ gene expression in kidney from a young (6 months) and an old (18 months) diversity outbred (DO) mouse showing genes that are ≥2-fold up/down-regulated. Dotted lines indicate twofold difference in expression. (**C**) Analysis of distribution shift and net expression change for the example shown in (B). Genes were sorted by expression level. AUC, area under curve. (**D**) Global gene expression changes in 192 DO mouse kidneys. Each row indicates each kidney; therefore, values (distribution shift, net expression change, and percent genes expression-changed) on the same horizontal line are from the same tissue. Kidneys were sorted by distribution shift of all genes within each age group. Affected (Af) and unaffected (Un) kidneys reflecting distinct physiological ages were determined with distribution shift of all genes as shown in the right bottom. (**E**) Microalbumin concentration in urine from DO mice with affected and unaffected kidneys. (**F**) Western blotting of heterochromatin marks and proteins in DO mouse kidneys. (**G**) Immunofluorescence staining of DO mouse kidneys for lamin B1 and heterochromatin mark (H3K9me2/3). White dotted lines indicate the boundaries of renal tubules. Error bars indicate SD of three replicates. *P < 0.05, **P < 0.01, and ***P < 0.001.

The three-dimensional (3D) organization of chromatin plays an important role in gene regulation. Each cell type in the body has a unique chromatin architecture optimized for the precise gene expression of that cell type (*1*, *2*). Heterochromatin, a condensed form of chromatin that silences transcription of associated genes, physically interacts with the nuclear lamina and inner nuclear membrane (INM) proteins, which form the inner face of the nuclear envelope. Thus, heterochromatin generally localizes toward the nuclear periphery and is spatially separated from its open counterpart, euchromatin, in the center of the nucleus (Fig. 1A).

Aging causes global disorganization of chromatin architecture. Disruption of nuclear lamina/INM proteins and decondensation of associated heterochromatin are common features of normative aging, cellular senescence, and premature aging diseases (fig. S1A and table S1A) (*3*–*7*). This disorganization of chromatin architecture has long been speculated to be the primary culprit behind age-associated physiological deterioration; however, there is no direct evidence for a molecular mechanism that might explain it.

Our recent studies have implicated CpG islands (CGIs) in chromatin architecture (*8*–*10*). CGIs are long (~1 kb) DNA elements in mammalian genomes with high frequencies of CpG dinucleotides compared to surrounding regions. In mammals, about 60% of all genes contain CGIs at their promoters (CGI^+^ genes); these genes are broadly expressed throughout the body. The remaining 40% of genes lack CGIs (CGI^−^ genes) and are expressed more specifically in particular tissues (*8*, *10*, *11*). CGI^+^ and CGI^−^ genes are regulated by distinct mechanisms (Fig. 1A) (*8*–*10*): CGI^−^ genes generally reside within nuclear lamina–associated heterochromatin when they are transcriptionally inactive and associate with euchromatin when they are transcriptionally active. By contrast, CGI^+^ genes reside in euchromatin regardless of whether they are active or repressed.

On the basis of these findings of a dual-mode gene regulation mediated by CGIs (Fig. 1A), we hypothesized that disruption of the nuclear lamina and/or associated heterochromatin during aging would specifically affect the transcriptional status of CGI^−^ genes, resulting in their uncontrolled expression. Here, we test this hypothesis using mouse models of normal and pathological aging and meta-analyses of published data from humans and mice to investigate the impact of disrupted chromatin architecture on CGI^−^ gene regulation. Our findings indicate that disorganization of nuclear architecture drives global CGI^−^ gene up-regulation during aging and, moreover, these changes in gene expression explain the chronic inflammation and other degenerative changes associated with aging.

## RESULTS

### Global up-regulation of CGI^−^ genes during aging

To understand how expression of CGI^+^ and CGI^−^ genes (table S2) changes during aging, we performed RNA sequencing (RNA-seq) of kidneys and hearts from diversity outbred (DO) mice. DO mice are a genetically diverse mouse resource that mimics the complexity of the human population with variable rates of physiological aging (*12*, *13*). Figure 1B shows an example of gene expression in kidneys from 18- and 6-month-old mice, where 33.71% of CGI^−^ genes were up-regulated over twofold in the aged kidney compared to the young kidney. In contrast, the corresponding proportion in CGI^+^ genes was less than 10%.

To systematically characterize this global misexpression of genes during aging, we monitored the significance (i.e., distribution shift, *P*) and directionality [i.e., net expression change, ΔAUC (area under the curve)] of expression changes of the entire CGI^+^ or CGI^−^ genes (see Materials and Methods). By analyzing the data in the example above (Fig. 1B), we observed global up-regulation of gene expression in the aged versus young kidneys (net expression change, >0), which was significant only in CGI^−^ genes (distribution shift; *P* = 5.49 × 10^−8^) (Fig. 1C). We then performed the same analysis across 192 kidneys and hearts of DO mice in three chronological age groups (6, 12, or 18 months old; *n* = 64 per group) using the median gene expression in young mice as a reference. The distribution of global gene expression was significantly shifted with age (fig. S1, B and C), but this shift was due mainly to the altered expression of CGI^−^ genes (*P* = 6.93 × 10^−7^ in kidneys and *P* = 1.27 × 10^−7^ in hearts) rather than CGI^+^ genes (*P* > 0.09 in both tissues). Furthermore, nearly all aged (18-month-old) kidneys and hearts of DO mice with significant distribution shifts showed up-regulated gene expression, especially of CGI^−^ genes (Fig. 1D, fig. S1D, and table S3). Up to 40% of CGI^−^ genes were up-regulated ≥2-fold in aged kidneys and hearts when compared to young tissues. These data indicate that CGI^−^ genes are frequently up-regulated during chronological aging.

Among DO mice of the same chronological age, we observed variable extents of distribution shift and CGI^−^ gene misexpression. Of the 64 18-month-old mice, for example, 17 individuals showed a significant distribution shift and CGI^−^ gene misexpression in kidneys, whereas 31 individuals did not (hereafter, we call these kidneys “affected” and “unaffected,” respectively; Fig. 1D and fig. S1, D and E; see also fig. S1, F and G). To understand the nature of this variability, we compared kidney function in the affected and unaffected individuals by measuring microalbumin concentration in urine. Mice with affected kidneys had a significantly higher incidence of renal dysfunction as evidenced by albuminuria compared to mice with unaffected kidneys (Fig. 1E). This indicates that CGI^−^ gene misexpression coincides with the physiological deterioration of kidney function during aging; thus, the variable extent of misexpression in the older age group reflects variable rates of physiological aging in the population. By Western blotting and immunofluorescence microscopy, we found that the affected kidneys had significantly lower levels of the nuclear lamina component lamin B1, the INM protein lamin B receptor (LBR), and the heterochromatin protein HP1α, when compared to young or unaffected aged kidneys (Fig. 1, F and G, and fig. S1H). Concomitantly, the heterochromatin marks H3K9me2/3 were significantly decreased in affected kidneys, while the marks for polycomb-mediated gene repression (H3K27me3) or euchromatic active promoters (H3K4me3) were not significantly affected (Fig. 1, F and G, and fig. S1, I and J). These results suggest that disorganization of the nuclear lamina/heterochromatin may be directly linked to global up-regulation of CGI^−^ genes during aging.

### Disorganization of nuclear architecture during aging drives CGI^−^ gene misexpression

**Fig. 2.**
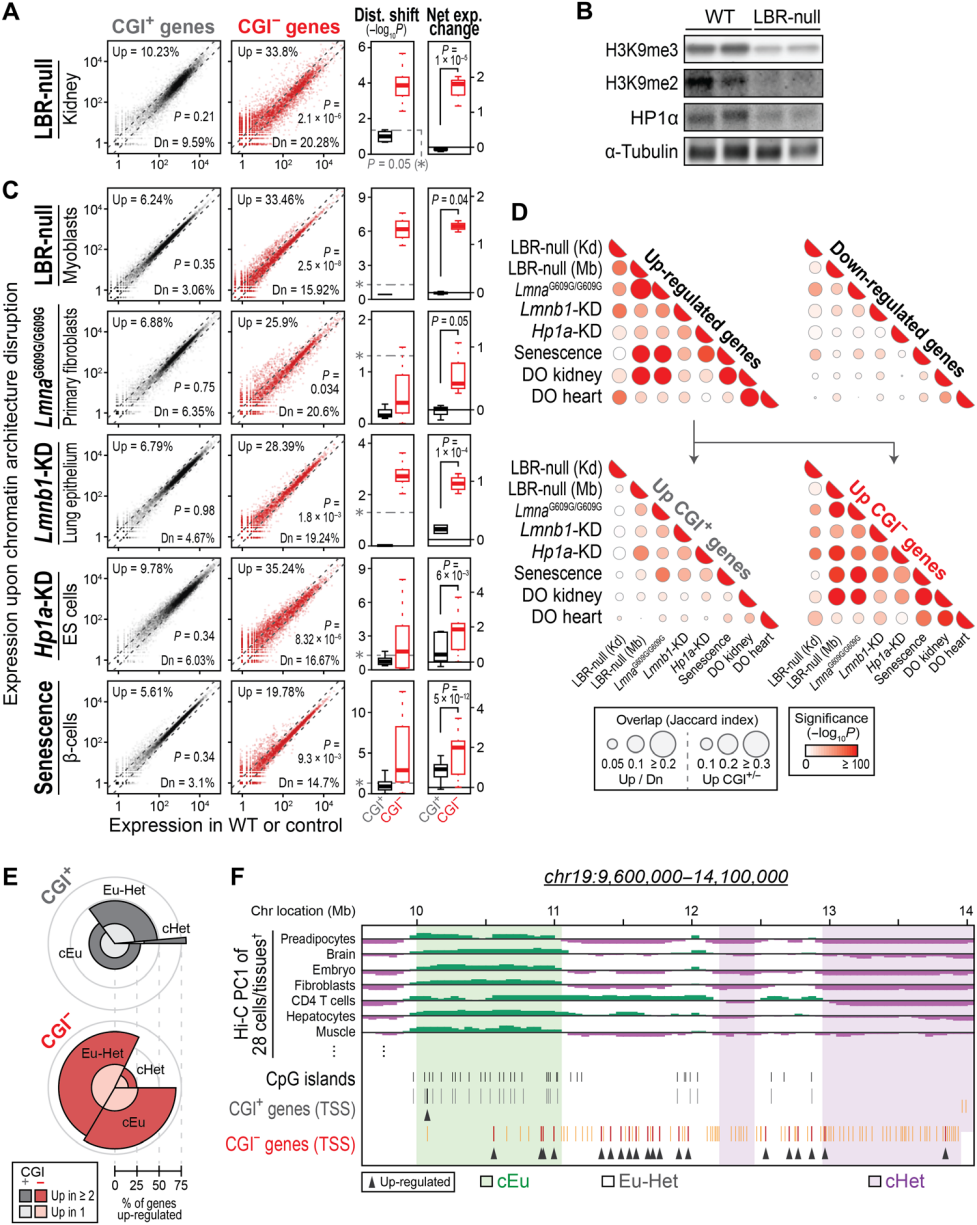
Disruption of nuclear architecture during aging drives uncontrolled expression of CGI^−^ genes. (**A** and **C**) Representative data showing expression (left), distribution shift, and net expression change (right) of CGI^+/−^ genes in LBR-null mouse kidneys (A) or in the context of chromatin architecture disruption (C). For full data, see fig. S2D and table S4. (**B**) Western blot of heterochromatin marks/component in LBR-null mouse kidneys. (**D**) Overlaps among expression-changed genes upon nuclear lamina/heterochromatin disruption or during aging. Significance was calculated by permutation tests (*n* = 1000). Kd, kidneys; Mb, myoblasts. (**E**) Portion of genes within three chromosomal domains that are up-regulated upon nuclear lamina/heterochromatin disruption or during aging [i.e., the eight contexts shown in (D)]. The polar angle of each group indicates its proportion of all CGI^+^ or CGI^−^ genes, while the radius indicates the up-regulated percentage within each group. Recurrently up-regulated genes in at least two conditions of the eight contexts shown in Fig. 2D are shown in darker gray/red colors. (**F**) A representative genomic landscape of CGI^+/−^ genes misexpressed during aging. †All Hi-C PC1 values of 28 cell/tissue types in this view and additional examples are illustrated in fig. S3.

To test whether CGI^−^ gene misexpression is a direct result of disruption of chromatin architecture during aging, we used mice with a loss-of-function mutation in the gene encoding LBR (*Lbr*^ic-J/ic-J^; hereafter, LBR-null mice; fig. S2A) (*14*, *15*). LBR is an INM protein that tethers heterochromatin to the nuclear envelope (Fig. 1A) (*16*, *17*); it is present in reduced amounts in various forms of aging (fig. S1, A and H, and table S1A). Kidneys from LBR-null mice showed clear signs of CGI^−^ gene misexpression (Fig. 2A) and loss of heterochromatin marks/components (Fig. 2B; see also fig. S2, B and C) compared to wild-type (WT) littermates. This implies that disorganization of nuclear architecture due to loss of LBR is sufficient to recapitulate the age-associated CGI^−^ gene misexpression and heterochromatin decondensation observed in DO mice.

We tested our hypothesis further by meta-analysis of published RNA-seq data generated from conditions that mimic the disruption of nuclear/chromatin architecture during aging (table S4) (*5*, *15*, *18*–*20*). Disruption of LBR, lamin B1, or HP1α directly triggered global up-regulation of CGI^−^ genes (Fig. 2C and fig. S2D). Consistent with this finding, we saw up-regulation of CGI^−^ genes also in senescent cells and upon mutation of the lamin A gene associated with Hutchinson-Gilford progeria syndrome (HGPS; *Lmna^G609G/G609G^*), both of which disrupt nuclear/chromatin architecture. Furthermore, reduction of the mutant form of lamin A (progerin) by systemic administration of CRISPR-Cas9 components (*5*) suppressed CGI^−^ gene misexpression in progeria (HGPS) mice (fig. S2E), suggesting a direct link between disruption of chromatin architecture and misexpression of CGI^−^ genes.

Moreover, the CGI^−^ genes that were up-regulated upon disruption of nuclear architecture largely overlapped with those that were misexpressed during normative aging (i.e., in the kidneys and hearts of DO mice) and in progeria/cellular senescence (Fig. 2D). This suggests that the up-regulation of CGI^−^ genes in each of these contexts was driven by the same trigger, i.e., disruption of nuclear/chromatin architecture. Together, we conclude that global misexpression of CGI^−^ genes during aging is attributable to age-associated nuclear/chromatin architecture disruption.

### Genomic landscapes of CGI^−^ genes misexpressed during aging

The data described above indicate that not all CGI^−^ genes are simultaneously up-regulated during aging: Some are more commonly misexpressed than others in cells that have disrupted nuclear/chromatin architecture. To investigate what determines this misexpression of some CGI^−^ genes but not others, we tested whether it is affected by chromosomal gene organization, which provides information on the spatial positioning of the genes in the nucleus and their epigenetic regulation (*8*–*10*, *16*, *17*). We collected Hi-C chromatin conformation data generated from 28 healthy, young adult mouse tissues and cells (*n* = 191; table S5A) and performed principal components analysis (PCA) to classify genomic regions in each sample as heterochromatin or euchromatin [represented by low or high PC1 values, respectively (*21*)]. By combining these data, we categorized the mouse genome into three domains: those that constitutively form euchromatin (cEu) or heterochromatin (cHet) and regions that may be euchromatin or heterochromatin depending on the cell or tissue type (Eu-Het) (fig. S3 and table S5B). Analysis of H3K9me2/3 ChIP-seq (chromatin immunoprecipitation followed by parallel sequencing) data generated from normal tissues indicated that CGI^−^ genes in all these three domains are more strongly associated with heterochromatin than CGI^+^ genes in the same domains (fig. S4 and table S6A), suggesting that heterochromatin decondensation during aging would affect CGI^−^ genes across all three domains. However, CGI^−^ genes in cHet domains, which are most strongly associated with heterochromatin, are rarely misexpressed upon disruption of nuclear architecture or during aging compared to those within cEu or Eu-Het domains (*P* < 2.2 × 10^−16^; Fig. 2, E and F; see also fig. S3). We further validated this unexpected result using nuclear lamina association and Hi-C compartment data generated from normal cells and tissues. Misexpressed CGI^−^ genes were generally located within euchromatic domains [i.e., non-LADs (lamina-associated domains) or A compartments] rather than within heterochromatic domains (i.e., LADs/B compartments) in all cell/tissue types analyzed (fig. S5, A to D). Consistent with genomic analysis, these genes were generally internalized rather than located at the nuclear periphery in both unaffected and affected kidneys (fig. S5E). All these data indicate that CGI^−^ genes in euchromatic domains, which associate more weakly with heterochromatin than those in broad heterochromatic domains, are more susceptible for activation upon chromatin architecture disruption and during aging.

The fact that CGI^−^ genes in heterochromatic domains are rarely activated during aging suggests that loss of heterochromatin is not sufficient to activate all associated genes simultaneously but rather requires additional layers of regulation. As our prior studies demonstrated that CGI^−^ genes are generally regulated by local binding of transcription factors (TFs) (*8*–*10*), we further performed TF-binding motif analysis. Notably, known TF-binding motifs were significantly more prevalent in the promoters of CGI^−^ genes in euchromatic domains when compared to those in cHet domains (*P* < 2.2 × 10^−16^ for both), and this was even more evident in recurrently up-regulated CGI^−^ genes (fig. S5F and table S7). Together, our data suggest that the recurrent misexpression of CGI^−^ genes in euchromatic domains is due to their increased susceptibility to uncontrolled TF-mediated activation in aged nuclei with decondensed heterochromatin.

### CGI^−^ gene misexpression causes loss of cellular identity during aging

Next, we questioned how CGI^−^ gene misexpression contributes to degenerative changes during aging. We first focused on the fact that precise heterochromatin formation is critical for maintaining cellular identity (*22*, *23*). As CGI^−^ genes are generally expressed in tissue/cell type–limited manners (*8*, *11*), we reasoned that heterochromatin decondensation and the resulting uncontrolled expression of CGI^−^ genes might drive the loss of cellular identity that has been observed in various aged mammalian tissues (*24*–*26*).

**Fig. 3.**
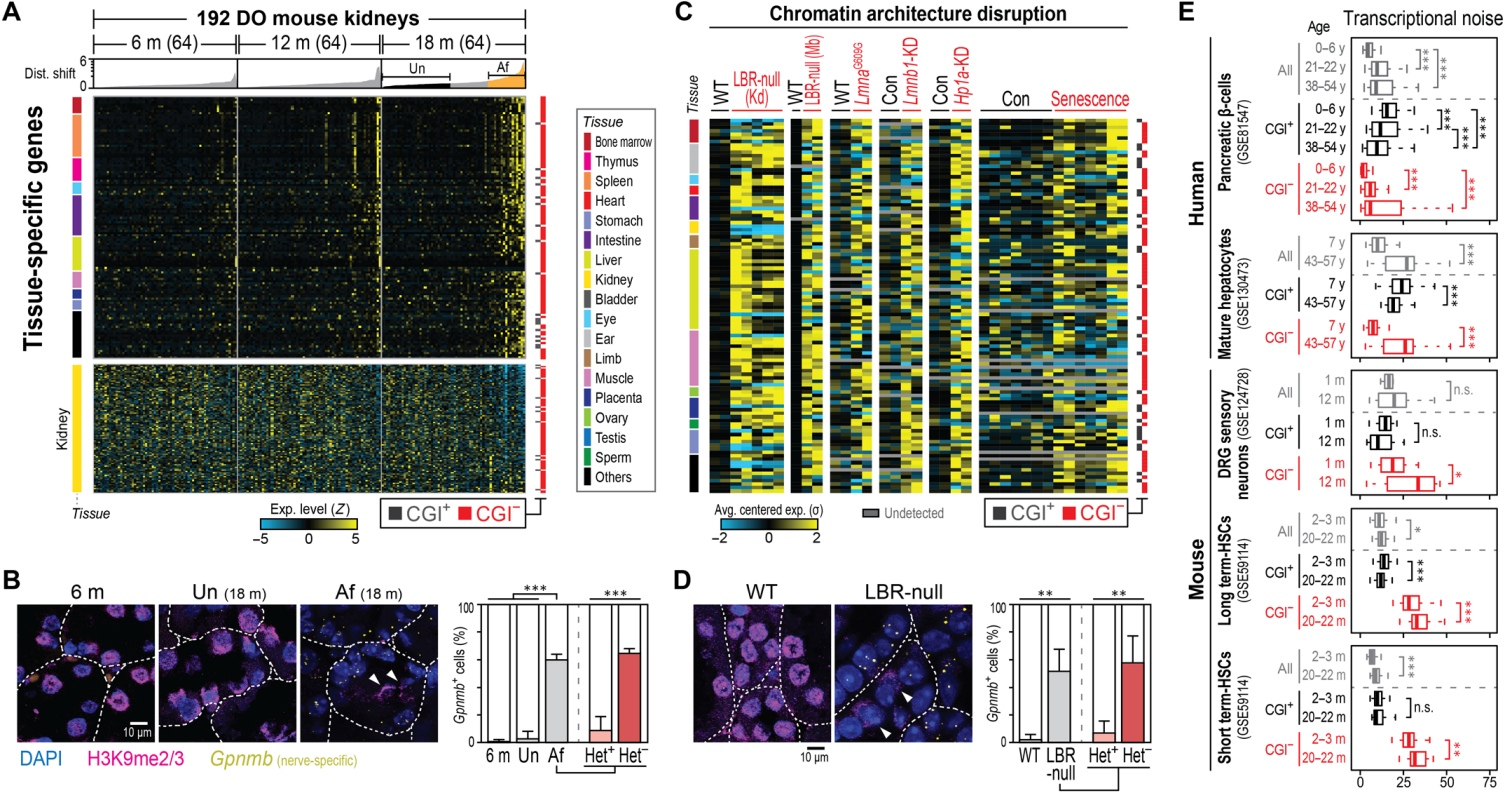
CGI^−^ gene misexpression causes loss of cellular identity during aging. (**A** and **C**) Expression levels of tissue-specific genes in DO mouse kidneys (A) or upon chromatin architecture disruption (C). Shown are tissue-specific genes that are significantly up/down-regulated in affected kidneys [versus unaffected, false discovery rate (FDR) < 0.05; top part of (A)], kidney-specific genes [bottom part of (A)] or upon nuclear architecture disruption (versus controls, at least threefold in three or more contexts) (C). For a full list of the genes, see table S9 (A and H). Un, unaffected; Af, affected kidney. (**B** and **D**) mRNA-FISH (*Gpnmb*) and immunofluorescence (H3K9me2/3) assays in kidneys from DO (B) or LBR-null (D) mice. Note that nuclei with intact heterochromatin (white triangles; Het^+^) rarely misexpress nerve-specific CGI^−^ gene (*Gpnmb*). White dotted lines indicate the boundaries of renal tubules. Error bars indicate SD of four replicates. (**E**) Age-associated increase in transcriptional noise (table S10). DRG, dorsal root ganglion; HSC, hematopoietic stem cell. *P < 0.05; **P < 0.01; ***P < 0.001; and not significant (n.s.), *P* ≥ 0.05. DAPI, 4′,6-diamidino-2-phenylindole.

To test this hypothesis, we defined 2243 tissue-specific genes in 28 tissue types by analyzing RNA-seq data from 578 young, healthy adult mouse tissues (table S8A) and monitored their expression in DO mice (table S8B). As expected, affected kidneys from DO mice expressed various tissue-specific genes that were not typically expressed in young kidneys, including spleen-, intestine-, eye-, and liver-specific genes (Fig. 3A and table S9A). The majority of these (85.12%) were CGI^−^ genes. By contrast, the expression of kidney-specific genes was mildly decreased in affected kidneys. We confirmed these findings by performing mRNA–fluorescence in situ hybridization (mRNA-FISH) for tissue-specific genes normally not expressed in kidney (*Gpnmb*, *Csn3*, and *Krt20*; Fig. 3B and fig. S6A). These genes were transcribed in affected kidneys from DO mice, and this was most evident in cells lacking H3K9me2/3 (Fig. 3B), again suggesting a direct link between heterochromatin decondensation and CGI^−^ gene misexpression. We observed disrupted tissue-specific gene expression also in hearts from DO mice (fig. S6, B and C, and table S9, B and C) and when we analyzed using previously defined tissue-specific gene sets [National Center for Biotechnology Information (NCBI) UniGene and InterPro Uptissue; fig. S6, D to G, and table S9, D to G]. Moreover, these patterns were recapitulated upon nuclear lamina/heterochromatin disruption and in progeria/cellular senescence (Fig. 3, C and D; fig. S6, H and I; and table S9, H to J). We conclude from these findings that disorganization of chromatin architecture and the resulting uncontrolled expression of CGI^−^ genes disrupt tissue-specific gene expression programs, causing aged cells to lose their functional identities.

Recent studies using single-cell RNA-seq have indicated that aging increases cell-to-cell transcriptional variability (“transcriptional noise”), which leads to loss of cellular identity and impaired tissue homeostasis (*24*, *25*, *27*). To investigate the possible role of CGI status on this transcriptional noise in aging, we performed a meta-analysis of published single-cell RNA-seq data for various types of human and mouse cells (table S10). Consistent with previous reports, the transcriptional noise of all genes generally increased during aging in both human and mouse cells (Fig. 3E). When the data for CGI^+^ or CGI^−^ genes were analyzed separately, transcriptional noise increased significantly during aging only in CGI^−^ genes; transcriptional noise in CGI^+^ genes either did not change or decreased slightly. These findings indicate that the increased transcriptional noise observed in various aged tissues (*24*, *25*, *27*) is driven by dysregulation of CGI^−^ genes rather than CGI^+^ genes.

### CGI^−^ gene misexpression is a major contributor to age-associated secretory phenotypes

**Fig. 4.**
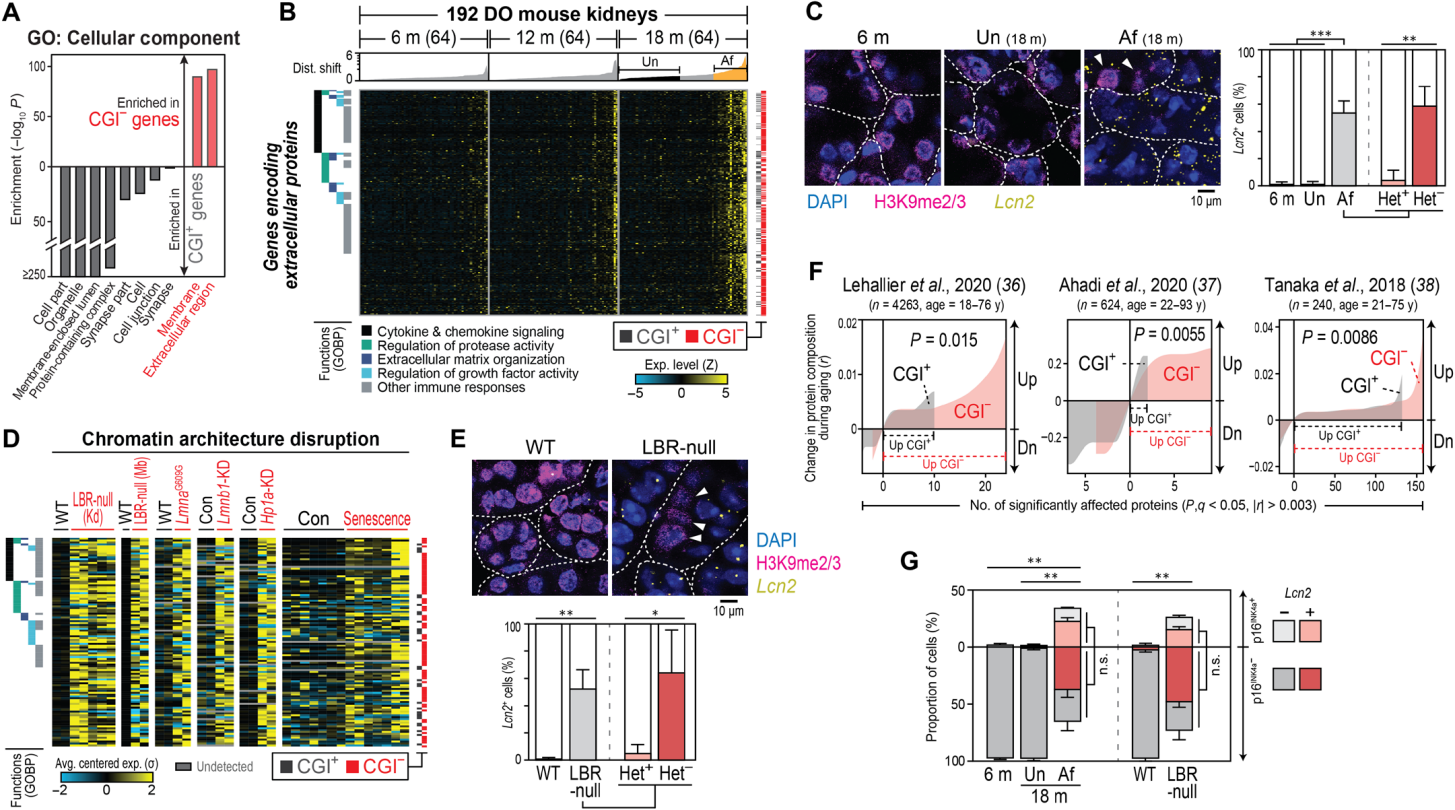
CGI^−^ gene misexpression drives age-associated secretory phenotypes. (**A**) GOCC analysis of all CGI^+/−^ genes. Annotations of CGI^+/−^ genes to depth-1 GOCC (the highest level in the GOCC hierarchy tree) are shown. *P* values were calculated using hypergeometric distributions. (**B** and **D**) Expression of genes encoding extracellular proteins in DO mouse kidneys (B) or upon chromatin architecture disruption (D). Shown are extracellular protein-coding genes that are significantly up/down-regulated in affected kidneys (versus unaffected, FDR < 0.05) (B) or upon nuclear architecture disruption (versus controls, at least threefold in three or more contexts) (D). For a full list of the genes and their curated functions (GOBP, GO Biological Process), see table S5 (A and C). (**C** and **E**) mRNA-FISH (*Lcn2*, a proinflammatory secretory CGI^−^ gene) and immunofluorescence (H3K9me2/3) assays in kidneys from DO (C) or LBR-null (E) mice. White dotted lines indicate the boundaries of renal tubules. Note that nuclei with intact heterochromatin (white triangles; Het^+^) rarely misexpress secretory CGI^−^ gene (*Lcn2*). (**F**) Meta-analysis of published plasma-proteome profiling data. (**G**) Portions of *Lcn2*-expressing cells among cells with and without senescence marker (p16^INK4a^ mRNA) expression. See also fig. S8 for the p16^INK4a^ mRNA and galactosidase β1 (GLB1) immunofluorescence staining. *P < 0.05, **P < 0.01, and ***P < 0.001. All error bars indicate SD of four replicates.

Another remarkable degenerative change during aging is uncontrolled secretory phenotype: A variety of aged mammalian cells and tissues [e.g., vascular endothelial cells (*28*), adipose tissues (*29*), bone (*30*), skin (*31*), and muscle (*32*)] express and secrete various extracellular proteins that disrupt intercellular communication and the tissue microenvironment. In particular, senescent cells enriched in aged tissues secrete various proteins that induce chronic inflammation both locally and systemically [known as the senescence-associated secretory phenotype (SASP)] (*33*, *34*). The upstream mechanisms that trigger these age-associated secretory phenotypes remain unknown. We noticed that of the misexpressed CGI^−^ genes that we identified in DO mouse kidneys (Fig. 1), nearly a quarter (23.23%) encoded secreted proteins, whereas only 8.33% of misexpressed CGI^+^ genes did. Moreover, gene association analysis using the Cellular Component sub-ontology of Gene Ontology (GOCC) indicates that the protein products of CGI^+^ and CGI^−^ genes have distinct cellular locations (Fig. 4A): CGI^+^ genes generally encode intracellular proteins, whereas CGI^−^ gene products are enriched for extracellular proteins (*P* = 1.7 × 10^−97^) and plasma membrane–associated proteins (*P* = 2.7 × 10^−90^). This indicates that many CGI^−^ genes encode proteins that pass through the secretory pathway, suggesting that their global up-regulation might result in the secretory phenotypes seen during aging.

The affected kidneys (Fig. 4B and table S11A) and hearts (fig. S7A and table S11B) of DO mice misexpressed hundreds of genes encoding secreted proteins, and the majority of them were CGI^−^ genes (72.2% in kidneys and 87.63% in hearts). These secreted proteins included cytokines, chemokines, growth factors, proteases, and extracellular matrix components, reminiscent of the mixture of factors secreted in the SASP (*33*, *34*). Tissue staining for mRNA also indicated that renal tubular cells within affected kidneys from DO mice actively transcribed proinflammatory secretory CGI^−^ genes (*Lcn2*, *Cxcl13*, and *Il6*; Fig. 4C and fig. S7, B and C). Without exception, this misexpression of secretory protein-coding genes was recapitulated in cells in which nuclear/chromatin architecture was disrupted and in progeria and cellular senescence (Fig. 4, D and E, and table S11C). Furthermore, kidneys from affected DO mice and LBR-null mice showed signs of local inflammation, including leukocyte infiltration (CD45^+^; fig. S7D) and increased expression of genes encoding markers of inflammation (e.g., *Il1b*, *Il6*, and *Ifng*; table S3A) when compared to controls. These findings indicate that disruption of nuclear architecture and the resulting CGI^−^ gene up-regulation fuel chronic inflammation in aged tissues.

Our conclusions from the transcriptome analysis are also supported at the protein level. Large proportions of the recently profiled SASP proteins (*35*) are the products of CGI^−^ genes that are misexpressed in aged kidney and heart tissue from DO mice and upon disruption of nuclear/chromatin architecture (fig. S7E). Furthermore, meta-analysis of published plasma proteome data (*36*–*38*) revealed that most proteins whose levels increase in aged plasma are encoded by CGI^−^ genes (Fig. 4F). Recent reports have demonstrated that aged plasma can cause systemic inflammation and physiological deterioration of the entire body (*39*–*41*). Thus, our finding suggests that CGI^−^ gene misexpression in aged organs not only induces local effects but may also exert systemic detrimental effects. This is consistent with our data showing that levels of CGI^−^ gene misexpression in DO mouse kidneys correlate positively with those in hearts (fig. S7F). Together, our findings indicate that disruption of nuclear/chromatin architecture and resulting CGI^−^ gene misexpression during aging promote secretory phenotypes that locally and systemically fuel chronic inflammation and physiological deterioration.

Again, aged tissues have a larger proportion of senescent cells than young tissues. We thus tested whether CGI^−^ gene misexpression is a specific property of senescent cells or whether this occurs more generally in the cells of aged tissues regardless of their state of senescence. We stained kidneys from DO mice for two markers of cellular senescence: p16^INK4a^ mRNA and GLB1 protein (galactosidase β1, also known as senescence-associated β-gal). Affected DO mouse kidneys were significantly enriched with cells expressing these senescence markers (Fig. 4G and fig. S8). Misexpression of CGI^−^ genes was widespread in the kidney tubular cells, however, regardless of whether the cells expressed senescence markers (*P* > 0.2 in all cases). Instead, it coincided more significantly with the loss of heterochromatin marks (Figs. 3, B and D, and 4, C and E; *P* < 0.04 in all cases). This suggests that misexpression of CGI^−^ genes encoding secreted proteins during aging is due primarily to heterochromatin decondensation rather than to cellular senescence.

### CGI^−^ gene misexpression is a shared trait of mammalian aging and age-associated diseases

**Fig. 5.**
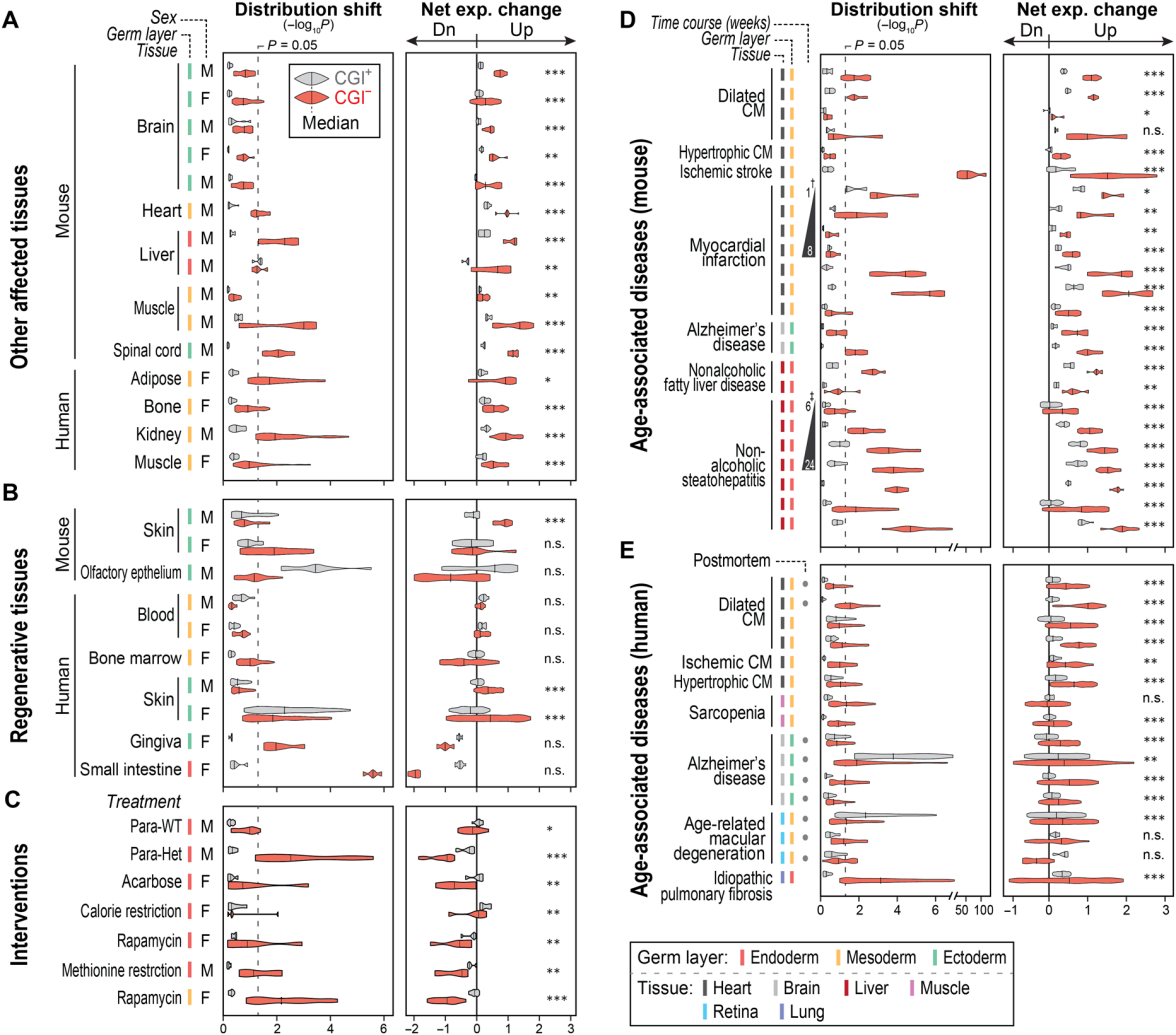
CGI^−^ gene misexpression is a common feature of aging and age-associated diseases. (**A** to **E**) Distribution shift and net expression change of CGI^+/−^ genes are shown in other age-affected tissues (A), in regenerative tissues (B), upon anti-aging interventions (C), and in age-associated diseases in mouse (D) and human (E). All RNA-seq datasets used in these figures are listed in table S12. †Note that severe CGI^−^ gene misexpression upon the surgery (at 3 months of age) was gradually ameliorated over the recovery period. ‡The level of CGI^−^ gene misexpression gradually increased along with disease progression (mediated by continued high fat diet). CM, cardiomyopathy; Para-WT or Para-Het, parabiosis of old mouse and WT young or mesencephalic astrocyte–derived neurotrophic factor–heterozygous young mouse, respectively. *P < 0.05; **P < 0.01; ***P < 0.001; and n.s., *P* ≥ 0.05.

To expand our findings, we tested whether misexpression of CGI^−^ genes is a feature of other aged tissues. We analyzed published, high-quality RNA-seq data from various normal young (10 to 30 years in human and 1 to 6 months in mouse) and old (≥50 years in human and ≥12 months in mouse) tissues (*n* = 1193; table S12; see Materials and Methods). We found significant up-regulation of CGI^−^ genes in many aged tissues, including brain, heart, muscle, and kidney from humans and mice, compared to the equivalent young tissues, irrespective of the sex of the animal or the germ layer origin of the tissue (Fig. 5A). Some tissues did not show CGI^−^ gene misexpression upon aging, however (Fig. 5B and fig. S9A); these included cells/tissues with a high turnover rate, which continually regenerate throughout life to replace dead or damaged cells, such as blood cells, skin, and olfactory and intestinal epithelial cells (*42*–*46*). These findings indicate that CGI^−^ gene misexpression occurs most commonly in aged cells within nonregenerative tissues rather than in those within regenerative tissues. Nevertheless, it is evident that global misexpression of CGI^−^ genes is a common feature across a wide variety of aged tissues.

To investigate whether anti-aging interventions might modulate CGI^−^ gene misexpression during aging, we analyzed RNA-seq data generated upon interventions such as treatment with acarbose or rapamycin, caloric restriction, and heterochronic parabiosis (table S12). These interventions suppressed expression of CGI^−^ genes in aged mice (Fig. 5C and fig. S9, B and C), especially for those genes that are misexpressed upon disruption of nuclear architecture and during aging (fig. S10A). These data suggest that age-associated CGI^−^ gene misexpression might serve as a novel biomarker to aid in the development of therapeutic strategies to ameliorate or delay age-associated degenerative changes.

Our data from DO mice indicated that CGI^−^ gene misexpression is linked with kidney failure (Fig. 1E). To investigate further the possible link between CGI^−^ gene regulation and age-related diseases, we performed a large-scale meta-analysis of published RNA-seq data from well-defined, noncancerous, age-associated diseases (*n* = 13,673; table S12) (*47*, *48*). CGI^−^ genes were severely misexpressed in tissues from patients with various age-associated diseases (i.e., myocardial, neurodegenerative, hepatic, and macular diseases) when compared to age-matched healthy controls (Fig. 5, D and E). Genes up-regulated in these age-associated diseases significantly overlapped with those misexpressed during normative aging or upon disruption of chromatin architecture (fig. S10A), and most of these were CGI^−^ genes in euchromatic domains (cEu and Eu-Het; fig. S10B). This indicates that these CGI^−^ genes were probably misexpressed in age-associated diseases for the same reason as in normative aging, i.e., due to disrupted nuclear/chromatin architecture. Together, our integrative analyses demonstrate that CGI^−^ gene up-regulation is a fundamental trait of mammalian aging, and its gradual progression may ultimately give rise to diseases associated with aging.

## DISCUSSION

Disruption of the nuclear lamina and associated heterochromatin has been described in various normal and pathological forms of aging for many years, yet its impact on gene expression and age-associated degenerative changes has remained unclear. In this study, we tested our hypothesis that the disruption of the nuclear lamina and associated heterochromatin seen during aging specifically affects the transcriptional homeostasis of genes that lack CGIs (CGI^−^ genes), which generally associate with heterochromatin at the nuclear periphery. We show that, indeed, this is the case: in humans and mouse models, global up-regulation of CGI^−^ gene expression is a hallmark of normal and pathological aging. CGI^−^ gene misexpression plays a central role in age-associated degenerative changes by penetrating and interconnecting previously established hallmarks of aging (*49*): Disruption of nuclear architecture and epigenetic alterations in aged or senescent cells are tightly associated with CGI^−^ gene up-regulation, which, in turn, disturbs intercellular communication. Moreover, CGI^−^ gene misexpression provides insights into the underlying molecular mechanisms of various phenomena observed in aged cells, including global loss of functional identity and increased transcriptional noise. In particular, a large fraction of the misexpressed CGI^−^ genes encode secreted proteins, many of which are associated with the SASP; aged kidneys and hearts from DO mice, mouse models with disrupted nuclear architectures, and progeria and senescent cells express proinflammatory secretory CGI^−^ genes, and most proteins whose levels increase in aged plasma are encoded by CGI^−^ genes. Together, our findings suggest that disorganization of the nuclear periphery in aged cells results in misexpression of CGI^−^ genes that are a direct source of systemic inflammatory mediators associated with aging.

Another remarkable observation here is that genes lacking CGIs, especially those within specific domains of chromosomes (cEu and Eu-Het), are recurrently misexpressed during aging. One question that naturally arises is how euchromatic CGI^−^ genes are up-regulated upon loss of heterochromatin. Significantly higher heterochromatin signals at CGI^−^ genes within these broad euchromatic domains (cEu and Eu-Het) than at CGI^+^ genes within the same domains (figs. S3 and S4) indicate that these genes are still regulated by heterochromatin despite exhibiting weaker heterochromatin association compared to those in broadly heterochromatin forming CGI^−^ genes (i.e., within cHet domains). This is supported by prior studies demonstrating that local heterochromatin formation within euchromatic domains can suppress expression of surrounding genes [e.g., “local heterochromatin” (*50*), “restricted heterochromatin” (*51*), or “focal heterochromatin” (*52*, *53*)] and that up-regulated CGI^−^ genes located within euchromatic domains, such as inflammatory genes *Tnf* and *Il6*, are regulated by heterochromatin formation (*54*, *55*). Our counterintuitive observation also explains, at least in part, why heterochromatin decondensation during aging has not been clearly linked with gene expression changes in prior studies (*56*, *57*).

The distribution of genes along with chromosomes is not random but rather optimized for precise gene regulation and function (*9*, *58*). CGI^−^ genes in euchromatic domains (i.e., cEu and Eu-Het) that are frequently misexpressed in nonimmune cells such as kidney tubular cells (fig. S7C) are generally enriched with secretory factors (fig. S10C) involved in the innate immune response (fig. S10D; see also table S7B). In alignment with recent studies showing that nonimmune cells obtain immune cell-like characteristics during aging (*59*, *60*), our results suggest that these genes may have evolved to acquire chromosome positions that enable rapid and simultaneous activation upon injury or infection to recruit immune cells (i.e., normal inflammation). From this perspective, our data can be interpreted in another way: Under normal conditions, heterochromatin safeguards against misexpression of inflammatory CGI^−^ genes located in euchromatic domains, whereas in aging, nuclear architecture disruption and heterochromatin decondensation result in uncontrolled expression of these genes, fueling the chronic inflammation during aging known as inflammaging.

Note that various age-associated diseases feature CGI^−^ gene misexpression, suggesting that age-associated CGI^−^ gene misexpression may serve as a valuable, novel biomarker of physiological aging. Further study to clarify the direct link between dysregulation of CGI^−^ genes and age-associated disease should yield greater insight into the pathogenesis of those diseases and identify potential therapeutic targets for delaying or ameliorating age-associated degenerative changes.

## MATERIALS AND METHODS

### Animals and sample collection

DO mice across three age groups were prepared as previously described (*12*, *13*). Briefly, an initial cohort consisting of 600 DO mice (300 males and 300 females) were bred at the Jackson Laboratory (stock number 009376) across five breeding waves (representing generations 8 to 12). Subsequently, 100 males and 100 females were randomly assigned to each of three groups at 6, 12, and 18 months of age. In this study, 192 mice were randomly selected with approximately equal representation of both sexes and age groups for sample collection (64 mice for each age group; female/male = 34/30 in 6-month group, 32/32 in 12-month group, and 30/34 in 18-month group).

Heterozygous LBR-null mice were recovered from cryopreservation at the Jackson Laboratory (C57BL/6J-*Lbr*^ic-J^/J; stock number 000529). After breeding and genotyping, three homozygous LBR-null mice (*Lbr*^ic-J/ic-J^) and WT littermates were randomly selected and sacrificed at 4 weeks of age.

All mice were maintained on standard rodent chow diet (5K52, LabDiet) in a pathogen-free animal room with a set temperature ranging from 20° to 22°C and a 12-hour light/12-hour dark cycle. Upon being sacrificed, the right kidneys of both DO and LBR-null mice and total hearts of DO mice were flash-frozen, then pulverized, and aliquoted for RNA and protein extraction. Urine samples of DO mice were collected at the selected ages, and urinary albumin was measured on a chemistry analyzer (Beckman Coulter AU680). The left kidneys were fixed in 4% paraformaldehyde, followed by embedding in paraffin and sectioning in 6-μm sections for tissue imaging. The Jackson Laboratory’s Institutional Animal Care and Use Committee approved all mouse experiments involved in this study (AUS no. 06005 for DO mice and AUS no. 16034 for LBR-null mice).

### RNA isolation and RNA-seq

Kidneys and hearts of DO mice were processed as follows. The pulverized samples were lysed and homogenized in Ambion TRIzol reagent (15596026, Thermo Fisher Scientific). Total RNA was isolated using miRNeasy Mini Kit (217004, QIAGEN) according to the manufacturer’s protocol with the optional deoxyribonuclease digest step. Polyadenylate [poly(A)] + RNA-seq libraries were generated using the TruSeq Stranded mRNA Library Prep Kit v2 (RS-122-2001, Illumina). Libraries were pooled and sequenced 100–base pair (bp) single-end on the HiSeq 2500 (Illumina) using TruSeq SBS Kit v4 reagents (Illumina) at the Jackson Laboratory (Bar Harbor, ME).

The LBR-null kidneys were lysed and homogenized in TRI Reagent (R2050, Zymo Research). TRIzol RNA Miniprep Plus Kit (R2070, Zymo Research) was used to isolate total RNA. Library preparation with poly(A) selection and 150-bp paired-end sequencing on an Illumina HiSeq 2500 were performed by GENEWIZ (South Plainfield, NJ).

### Definition of CGI^+^ and CGI^−^ genes

CGI^+^ and CGI^−^ genes were defined as previously described (*8*–*10*) with minor modifications. Briefly, we combined two methods of CGI annotation to minimize noise: (i) CGIs defined by sequence characteristics [i.e., GC content, ≥50%; length, >200 bp; and CG_observed_/CG_expected_, >0.6; downloaded from the UCSC database (http://genome.ucsc.edu/)] (*61*) and (ii) experimentally validated CGIs identified by CxxC-affinity purification followed by parallel sequencing (*62*). For experimentally validated CGIs, CxxC affinity–purified regions in the sperm, blood, and cerebellum (both in mouse and human) were identified using model-based analysis of ChIP-seq (MACS) 1.4.2 (*63*) with a *P* value cutoff of 1 × 10^−5^, and non–tissue-specific consensus CxxC-domain binding regions were selected and used. Then, genes with transcription start sites (TSS) surrounded by both consensus CxxC binding regions and UCSC-downloaded CGIs (±500 bp) were defined as CGI^+^ genes, while genes not associated with both elements from 500 bp upstream of TSS through the gene body were defined as CGI^−^ genes. All CGI^+^ and CGI^−^ genes used in this study are listed in table S2 for both mouse and human.

### Transcriptome analysis

For DO mice, raw RNA-seq data were aligned as described previously (*12*, *13*). Briefly, Genotyping By RNA-Seq software (https://gbrs.readthedocs.io/en/latest/) was used to align RNA-seq reads and to reconstruct the individual haplotypes of DO mice, and total expression levels were measured using Expectation-Maximization algorithm for Allele Specific Expression (*64*). Raw LBR-null kidney RNA-seq reads (FASTQ) were aligned to the mouse genome (mm9, NCBI Build 37) using STAR 2.6.0 (*65*). Sequencing data for each LBR-null mouse sample with aligned read counts ≥2 × 10^7^ were used. The read count data from both experiments were normalized, and statistical tests were performed to determine differentially expressed genes (adjusted *P* value using Benjamini-Hochberg method, <0.05 and fold change, ≥2) using DESeq2 1.25.1 (*66*).

For meta-analyses of RNA-seq, all available RNA-seq data deposited in the Gene Expression Omnibus (GEO) as of 19 March 2020 were included in this study. RNA-seq data were downloaded from NCBI SRA, and FASTQ files were extracted using SRA Toolkit 2.5.5 (https://github.com/ncbi/sra-tools). Sequences were aligned to the mouse (mm9, NCBI Build 37) or human (hg19, GRCh37) genomes using STAR 2.6.0 (*65*), and read counts were normalized using DESeq2 1.25.1 (*66*) as above. Each dataset in our meta-analysis had a variable number of samples, and this resulted in a significant sample number bias while defining differentially expressed genes using DESeq2. To enable fair comparison of differentially expressed genes from each dataset (Figs. 2, 3C, and 4D), genes with ≥2-fold difference in average expression level were determined as differentially expressed genes.

To identify tissue-specific genes, we collected and analyzed 578 poly(A) + RNA-seq data generated from normal adult mouse tissues that we profiled previously (table S8A) (*9*). We used the Human Protein Atlas (*67*) criteria to define tissue-specific genes; genes whose expression levels are fivefold or higher in a specific tissue compared to any other tissues. These tissue-specific genes are listed in table S8B. Tissue-specific genes previously defined by “restricted expression” based on expressed sequence tag profile (“NCBI UniGene”) and those defined by text mining (“InterPro Uptissue”) were downloaded from the DAVID knowledgebase (*68*).

To investigate the age- and disease-associated expression patterns of CGI^+/−^ genes, available human and mouse tissue RNA-seq data generated in aging or related disease contexts were collected from all GEO-deposited RNA-seq data. Noncancerous age-related diseases were defined on the basis of literature reviews (*47*, *48*). The data were filtered to retain only studies satisfying all of the following criteria: (i) data generated from untreated samples (i.e., not treated with chemicals, hormones, cytokines, etc.), (ii) datasets having five or more samples in both the aged or young group, (iii) sequencing data for each sample with raw read counts ≥1 × 10^7^, and (iv) datasets with sex information. To minimize bias, we grouped the data by sex and monitored gene expression changes within each group. Genes on the Y chromosome were excluded in this analysis to avoid possible confounding effects of sex-related differences at the chromosome level. To compare young and old tissue gene expression patterns in human, the data generated from samples with age 10 through 30 years were categorized as “young,” while those with age 50 years or over, as well as postmortem tissue biopsies, were categorized into group “aged.” For mouse, data from 1- to 6-month-old tissues were categorized as young, and data from 12 months old or older were categorized as aged.

### Distribution shift and net expression change

Distribution shift was calculated with Wilcoxon Rank Sum test using “Statistics::Test::WilcoxonRankSum” package in Perl. Figure 1C shows that there is a clear difference in the expression patterns and its directionality between two samples (young versus aged). To geometrically define this difference, we calculated net expression change: We first scaled the data such that the *x* and *y* axes ranged from 0 to 10. Then, the AUC for each sample was calculated separately in the scaled plot, and net expression change was defined as the difference between the AUC values of a given pair of samples (e.g., young versus aged, control versus disease, etc.). Gene expression values were transformed to the log_10_ scale to avoid bias caused by a high random multiplicative error in high expression values. In short, net expression change was calculated using this formula

$$\begin{array}{c} \text{Net}\;\text{expression change}={\text{AUC}}^{\text{Aged}}-{\text{AUC}}^{\text{Young}}\\ =(\frac{\sum_{i=1}^{n}{\text{log}}_{10}{\text{Exp}}_{i}^{\text{Aged}}-\sum_{i=1}^{n}{\text{log}}_{10}{\text{Exp}}_{i}^{\text{Young}}}{n\times {\text{log}}_{10}{\text{Exp}}_{\text{max}}^{\text{Both}}}\;)\times 100 \end{array}$$

where *n* is the total number of genes, ${\text{Exp}}_{i}^{\text{Aged}}$ is the expression level of gene *i* in an aged sample, ${\text{Exp}}_{i}^{\text{Young}}$ is the expression level of gene *i* in a young sample, and Exp^Both^ = [Exp^Aged^, Exp^Young^].

While measuring distribution shift and net expression change, genes expressed in neither aged nor young samples were excluded. To avoid sex-related differences in whole-gene expression, females and males were analyzed separately. Affected and unaffected tissues of DO mice were determined by measuring distribution shift of all genes in each sample (distribution shift, >0.5 and <0.25, respectively) in comparison to the reference expression in the young group (median expression level across all young samples). Meta-analyses of published RNA-seq data were performed using all-pairs testing (*69*) with minor modifications. In detail, distribution shifts and net expression changes were calculated for each pairwise combination of two comparison groups [i.e., young versus aged (Fig. 5, A and B), control versus intervention (Fig. 5C), control versus disease (Fig. 5, D and E), and control versus nuclear architecture disruption (Fig. 2, A and C, and fig. S2D)]. If the mapped read count of one sample exceeds 1.5-fold of the other sample, then that pair was excluded from the further analysis to avoid any bias arising from read-depth differences. The significance of differences in net expression changes of CGI^+^ genes versus those of CGI^−^ genes was measured with one-tailed paired *t* test. Significance of gene set overlap was tested using permutation test (*n* = 1000). Codes for computing distribution shift and net expression change are freely available at https://doi.org/10.5281/zenodo.5579384.

### Hi-C data analysis

All available mouse Hi-C data deposited in NCBI GEO and 4D Nucleome (4DN; www.4dnucleome.org/) were listed on 19 March 2020. We then downloaded mouse Hi-C data generated from young, healthy, nonmeiotic, and nontreated mouse tissues or cells from NCBI SRA and 4DN (191 datasets; listed in table S5A). FASTQ files were extracted with SRA Toolkit 2.5.5 and aligned to mouse genome (mm9, NCBI Build 37) using Bowtie 2.3.2 (*70*). Hi-C analysis tools in Hypergeometric Optimization of Motif Enrichment (HOMER) suite 4.11.1 (*71*) were used for downstream analyses. For PCA, TSSs of housekeeping genes were used as marks for active chromatin (for “–active” option in “runHiCpca.pl” within HOMER). The mouse housekeeping genes were obtained from the Housekeeping and Reference Transcript Atlas (*72*).

To determine constitutively euchromatic (cEu) or heterochromatic (cHet), as well as interconvertible (Eu-Het) domains within the mouse genome, Hi-C data generated from the same cell or tissue types were gathered and only the data with the highest number of aligned reads was selected as the representative Hi-C data for the given cell/tissue type. Using 50-kb resolution, genomic region where at least 95% of cell/tissue types had either positive or negative Hi-C PC1 value was defined as cEu or cHet, respectively. All other regions were defined as Eu-Het, as shown in table S5B. The Y chromosome was excluded in this analysis to avoid any bias originated from sex differences.

### ChIP-seq and DNA adenine methyltransferase identification by high-throughput sequencing data analysis

All mouse ChIP-seq and DamID-seq (DNA adenine methyltransferase identification by high-throughput sequencing) data were listed on 19 March 2020 from NCBI GEO database. Among them, data generated from young, healthy, nonmeiotic, and nontreated mouse tissues were used for the subsequent analyses. FASTQ files were extracted with the SRA Toolkit version 2.5.5.

For ChIP-seq analysis, FASTQ files were aligned to the mouse reference genome (mm9, NCBI Build 37) using Bowtie 2.3.2 (*70*) with default options. Signal-based analyses were performed using duplicate filtered read pileup bedGraph files made from MACS 1.4.2 (*63*) with “–nomodel” and “–nolambda” options. We filtered high-quality H3K9me2/3 ChIP-seq data to exclude data containing high false-positive signals as previously described (*8*–*10*) with some modifications: (i) data with raw read counts ≥1 × 10^7^, (ii) data with ChIP signals detected in at least 99.9% of CGI^+/−^ genes, and (iii) data with the signal-to-noise ratio >0.01. Briefly, this ratio was computed from the bedGraph files for each ChIP-seq data using the following equation

$$\begin{array}{c} \text{Signal to noise ratio}=\\ \frac{\text{AUC}\;\text{of ChIP signals within peak regions}}{\text{AUC}\;\text{of ChIP signals outside of peak regions}} \end{array}$$

A total of 208 H3K9me2/3 ChIP-seq data met those criteria and were used in this study (table S6A). For DamID-seq analysis, the LADetector (version 8122016) (*73*) pipeline was used with default options (see table S6B for a full list of data). High-quality DamID-seq data with mapped read counts ≥1 × 10^7^ were used for downstream analyses. The Y chromosome was excluded in both ChIP-seq and DamID-seq analysis to avoid any bias originated from sex differences.

### Analysis of TF binding motifs

The number of known DNA motifs in the TSS-flanking region of each gene and its significance were measured using Analysis of Motif Enrichment 5.0.4 tool within the Multiple Expectation maximization for Motif Elicitation suite (*74*) with default parameters. False-positive motifs were excluded, and motifs with false discovery rate <0.05 were considered as significantly enriched motifs compared to other regions. The database of known motifs was downloaded from the HOMER Motif Database (“HOMER Known Motifs”) (*71*).

### GO analysis

To analyze cellular localization of gene products, GOCC terms at the first depth (the highest level in the hierarchy tree of GOCC) and its associated gene lists were obtained from Mouse Genome Informatics (www.informatics.jax.org/vocab/gene_ontology). For functional classification of genes, GOBP data were downloaded from BioMart of the Ensembl genome database (http://useast.ensembl.org/biomart/martview). Enrichment of CGI^+/−^ genes in GO terms was calculated using the hypergeometric distribution as we previously described (*8*–*10*). GO terms with *P* < 10^−6^ were determined as significantly enriched/depleted terms.

### Single-cell RNA-seq data analysis

We downloaded single-cell RNA-seq data from NCBI SRA along with author-provided read count tables from the NCBI GEO website (table S10). Cells with the detected genes <1000 or the read count number <1 × 10^5^ were not used for further analysis. We also excluded those if the number of individuals in each group is under 10. We used the age criteria that the original authors defined in their studies.

Transcriptional noise was determined as Enge *et al.* (*25*) previously described. Briefly, transcript counts were normalized into log-transformed counts per million (CPM), and the transcriptional noise was calculated with the following formula

Transcriptional noise = (1 – cor(*X_i_*, *Y*)) × 100

where cor is the Pearson correlation coefficient, *X_i_* is CPM of sample *i*, and *Y* is the average CPM of young samples.

### Western blotting

Right kidneys of DO mice and LBR-null mice were homogenized in T-PER Tissue Protein Extraction Reagent (78510, Thermo Fisher Scientific) with Pierce Protease Inhibitor Mini Tablets, EDTA-Free (A32953, Thermo Fisher Scientific). The tissue lysates were then centrifuged at 20,000*g* for 15 min, and the supernatant was collected, subjected to SDS–polyacrylamide gel electrophoresis, and transferred to a polyvinylidene difluoride membrane (GE Healthcare). Membranes were blocked with 3% bovine serum albumin (BSA) in tris-buffered saline containing 0.05% Tween 20 (TBST) at room temperature for 1 hour. Primary antibodies diluted in TBST were treated and incubated at 4°C overnight with the following conditions: rabbit anti-H3K9me2 (1:500; 07-441, Sigma-Aldrich), goat anti-HP1α (1:500; ab77256, Abcam), rabbit anti-H3K9me3 (1:1000; 49-1008, Thermo Fisher Scientific), and chicken anti–α-tubulin (1:3000; SAB3500023, Sigma-Aldrich). After washing with TBST, the membrane was incubated with species-specific horseradish peroxidase–conjugated secondary antibodies (1:10,000; Thermo Fisher Scientific) at room temperature for 1 hour. The signals were developed with the Pierce ECL chemiluminescent (Thermo Fisher Scientific) for 1 min.

### Immunofluorescence staining and mRNA/DNA-FISH

For immunofluorescence assays using paraffin-embedded tissues, tissue sections were rehydrated with xylene and ethanol. Antigen retrieval was conducted with incubation at 98°C for 30 min in citrate buffer [10 mM citric acid and 0.05% Tween 20 (pH 6.0)]. The sections were washed in phosphate-buffered saline (PBS); blocked with 5% BSA and 0.04% Triton X-100 in PBS for 1 hour; and stained with rabbit anti–Lamin B1 (1:100; ab133741, Abcam), mouse anti-LBR (1:100; ab232731, Abcam), rabbit anti-H3K4me3 (1:300; ab213224, Abcam), rabbit anti-H3K27me3 (1:300; 9733S, Cell Signaling Technology), and mouse anti-H3K9me2/3 (1:100; 5327S, Cell Signaling Technology) antibodies in PBS supplemented with 0.5% BSA and 0.04% Triton X-100 at 4°C overnight. After washing with PBS, the tissue sections were incubated with Alexa Fluor 488 (A-21206, Invitrogen), Alexa Fluor 594 (A-11005, Invitrogen), or Alexa Fluor 647 (A-31573, Invitrogen) antibodies (1:200 dilution) in PBS supplemented with 0.5% BSA and 0.04% Triton X-100 and counterstained with 4′,6-diamidino-2-phenylindole (DAPI; 10 μg/ml; 62248, Thermo Scientific). For actin cytoskeleton staining, Phalloidin Conjugates CF488A (1:40; 00042-T, Biotium) was applied after DAPI staining.

mRNA-FISH was performed on paraffin-embedded tissues using the RNAscope Multiplex Fluorescent Reagent Kit v2 (323100, Advanced Cell Diagnostics) and probes produced by the same manufacturer (RNAscope probe-Mm-Gpnmb-C3, 489511-C3; RNAscope probe-Mm-Lcn2-C3, 313971-C3; RNAscope Probe-Mm-Csn3-C2, 520671-C2; RNAscope probe-Mm-Krt20, 402301; RNAscope probe-Mm-Cxcl13, 406311; RNAscope Probe-Mm-Il6, 315891; and RNAscope Probe-Mm-Cdkn2a-tv2-C2, 447491-C2). Transcripts were detected following the manufacturer’s protocol (“Manual Fluorescent Assay on Formalin-fixed, Paraffin-embedded Samples”). According to the manufacturer’s recommendation, we used Opal 570 and Opal 690 (FP1488001KT and FP1497001KT, respectively, Akoya Biosciences) as fluorophores. When immunostaining and mRNA-FISH were combined, mRNA-FISH was performed following the protocol with the exception of the DAPI staining step. Tissue sections were then washed with PBS, and immunostaining was performed from the blocking step.

DNA-FISH was performed on paraffin-embedded tissues using the Oligopaint technique (*75*) with some modifications as previously described (*9*). Fluorescence-labeled FISH probe library was designed as single-stranded DNA 32-mers (ATTO-550) and synthesized by Arbor Bioscience (myTags Immortal probe libraries): A total of 19,722 oligos were prepared to detect eight loci harboring CGI^−^ genes that are up-regulated in affected kidneys [2465.25 ± 385.96 probes per locus; chr1:134450001–134620001 (*Nfasc*), chr1:139940001–140090001 (*Ptprc*), chr3:135700001–136000001 (*Bank1*), chr5:141330001–141490001 (*Card11*), chr10:28370001–28620001 (*Themis*), chr13:36940001–37160001 (*F13a1*), chr14:33760001–34010001 (*Wdfy4* and *Lrrc18*), and chr19:11470001–11690001 (*Ms4a4c*, *Ms4a4b*, *Ms4a6c*, *Gm8369*, *Ms4a6b*, *Ms4a4d*, and *Ms4a6d*)]. Staining was performed with 50-pmol probes in 2× Saline-Sodium Citrate containing Tween 20 (SSCT), 50% formamide, 30% dextran sulfate, and 10 μg of ribonuclease A. After staining, slides were washed and counterstained with DAPI (10 μg/ml; 62248, Thermo Scientific).

For imaging, fixed samples were prepared on slides with no. 1.5 coverslips. All images were collected with Zeiss Axio Imager M2 equipped with Zeiss ApoTome 2 and Plan-Apochromat 20×/0.8 M27 objective [for figs. S1 (I and J), S2 (B and C), and S7C] or Olympus FV1000 confocal on an inverted Olympus IX81 stand equipped with Plan Apochromatic 60×/1.42 numerical aperture oil emersion objective (for the other images). The microscopes were controlled with Zen Pro 3.1 or Olympus FluoView software, respectively. DAPI, Alexa Fluor 488, Alexa Fluor 594/Opal 570, and Alexa Fluor 647/Opal 690 were excited with the 405-nm laser line (50 mW LD), the 473-nm line (15 mW LD), the 559-nm line (15 mW LD), and the 635-nm laser line (20 mW LD), respectively. When using Zeiss Axio Imager M2, 16-bit images were captured with 2048 × 2048 pixel frame size (0.325-μm pixel size in *XY* and 0.5-μm pixel size in *Z*) with optical sectioning using Zeiss ApoTome 2. Using Olympus FluoView 1000, 12-bit images were captured with 1024 × 1024 pixel frame size (0.2-μm pixel size in *XY* and 1-μm pixel size in *Z*) with a frame average of two and with a Z-stack of the thickness of 10 μm.

Minimal image processing was applied before image analysis. Brightness and contrast were adjusted identically for compared image sets, and signal intensity was quantified using ImageJ Fiji 1.53c (https://imagej.net/software/fiji/). To measure signal intensity, the original size images were used with four replicates. To define marker/target expression in the cytosolic region of each cell, nuclear areas were enlarged as previously described (*76*) with minor modifications. Briefly, nuclear areas were selected automatically in the DAPI-stained images. As these selections do not encircle the cytosol, each selection was enlarged by 3 μm to include the cytosolic space using the “Enlarge” function in ImageJ Fiji. Thereafter, the fluorescence intensity within each cell body was measured independently. Markers or transcript signals located further than 3 μm from the nearest DAPI-positive region were excluded to minimize the noise from false-positive annotation. NEMO 1.5.1 (*77*) was used with default options to reconstitute 3D structure of nuclei and to determine localization of DNA-FISH signals. To display the images in the figures, 256 × 256 pixels or 341 × 341 pixels of original images were cropped to provide clear views of nuclei.

### Statistical analysis

Statistical analyses were performed using R software (v3.6.3), unless indicated otherwise in the method section for each analysis. Wilcoxon exact rank sum test was performed for statistical tests using “exactRankTests” package in R, as it is suitable for the data for possibly tied observations (https://cran.r-project.org/web/packages/exactRankTests), unless indicated otherwise in the method section and/or figure legend for each analysis. Figure legends contain replicate information. All data presented met the assumptions of the statistical test used. Box plots show median (line), quartiles (boxes), and range (whiskers). The other data are represented as each data point or mean and SD. Statistical significance is represented in each figure legend.

## Notes

View/request a protocol for this paper from *Bio-protocol*.
