# Supplementary material for: Misexpression of genes lacking CpG islands drives degenerative changes during aging
Source: Sci Adv. 2021 Dec 15;7(51):eabj9111. doi: 10.1126/sciadv.abj9111 (PMC8673774; doi:10.1126/sciadv.abj9111)
Supplement: Supplementary file 1 — Figs. S1 to S10 Legends for tables S1 to S12 [file sciadv.abj9111_sm.pdf]

Science Advances
AAAS

Supplementary Materials for

Misexpression of genes lacking CpG islands drives degenerative changes during aging

Jun-Yeong Lee, Ian Davis, Elliot H. H. Youth, Jonghwan Kim, Gary Churchill, James Godwin, Ron Korstanje, Samuel Beck*

*Corresponding author. Email: sbeck@mdibl.org

Published 15 December 2021, *Sci. Adv.* **7**, eabj9111 (2021)
DOI: 10.1126/sciadv.abj9111

The PDF file includes:

Figs. S1 to S10
Legends for tables S1 to S12

Other Supplementary Material for this manuscript includes the following:

Tables S1 to S12

A

	Normative aging	Senescence	Premature aging	
			Progeria (HGPS)	Werner syndrome
Cause	-	Telomere shortening, oncogene induction, etc.	*LMNA* mutation	*WRN* mutation
INM proteins	Loss of LAP2β, LBR	Loss of LBR, LAP2α, Emerin	Loss of LAP1, LAP2α, Emerin	Loss of LAP2β, LBR
Nuclear lamina proteins	Loss of Lamin A, Lamin B1	Loss of Lamin A, Lamin B1	Loss of Lamin B1, Progerin accumulation	Mislocalization of Lamin B1
Heterochromatin	Loss of H3K9me3, HP1α	Loss of H3K9me3	Loss of H3K9me3, HP1α	Loss of H3K9me3, HP1α

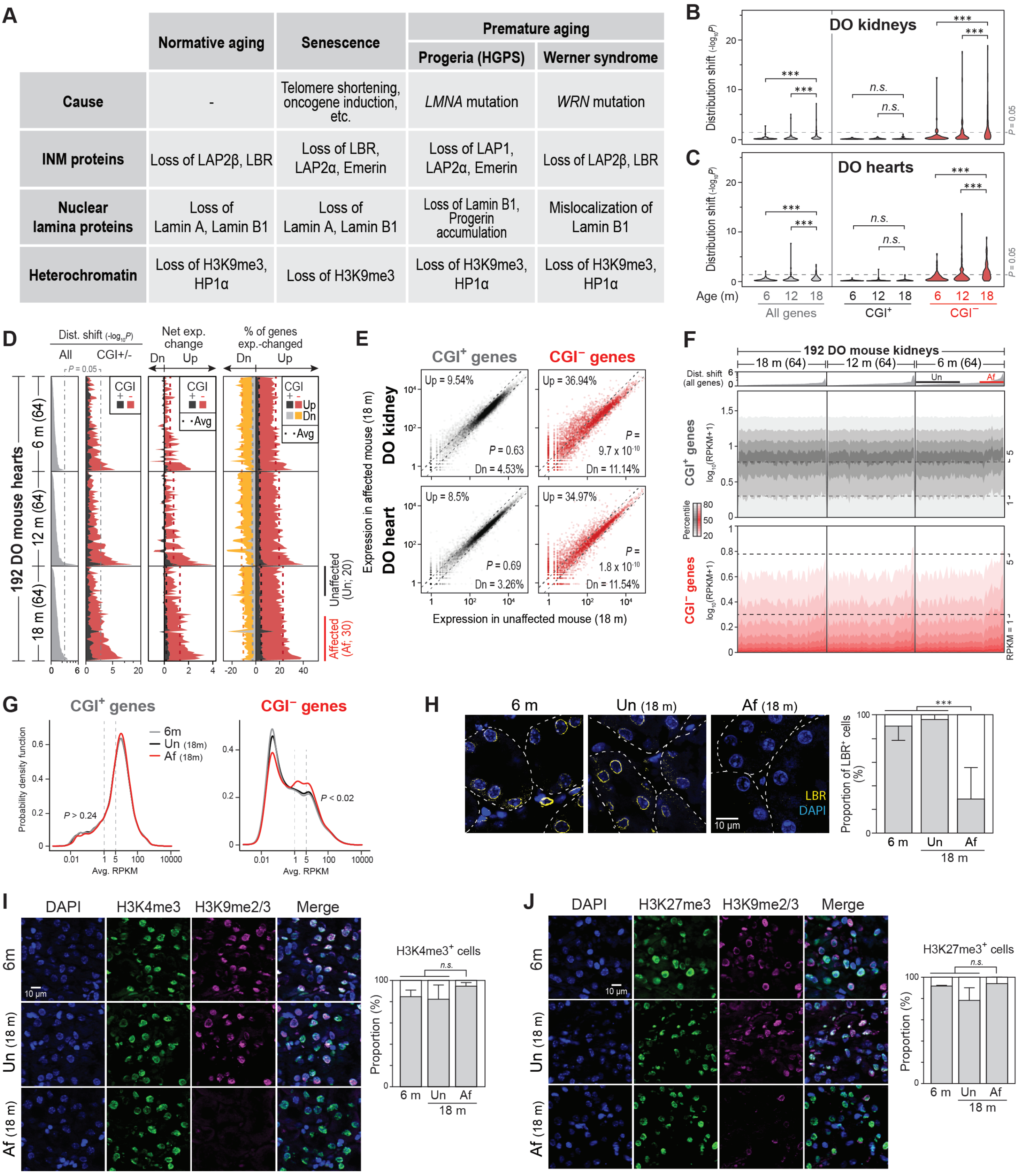

Fig. S1. Changes in gene expression within DO mice during aging. (A) Summary of defects in nuclear/chromatin architectures in various aging contexts. For references, see table S1. HGPS, Hutchinson-Gilford progeria syndrome. **(B and C)** Distribution shift during aging within kidneys (**B**) and hearts (**C**) of DO mice. **(D)** Global gene expression changes in 192 DO mouse hearts. Hearts were sorted by distribution shift of all genes within each age group. **(E)** Example comparison of CGI^{+} and CGI^{-} gene expression in kidneys or hearts from unaffected and affected DO mice. Dotted lines indicate 2-fold difference in expression. **(F and G)** Distribution of

expression level (RPKM) in DO mouse kidneys (**F**) and density plots showing average RPKM in 6 m, unaffected, and affected DO mouse kidneys (**G**). Although CGI^{-} genes have relatively low basal expression levels compared to CGI^{+} genes, many CGI^{-} genes were up-regulated during aging to expression levels that can result in physiological changes (i.e., RPKM > 1 or 5). **(H)** Immunofluorescence images of LBR in DO mouse kidneys. White dotted lines indicate the boundaries of renal tubules. **(I and J)** Immunofluorescence tissue staining combined with RNA-FISH in DO mouse kidneys. H3K4me3 (**I**) or H3K27me3 (**J**) were detected along with H3K9me2/3 and CGI^{-} gene *Lcn2* misexpressed upon disruption of chromatin architecture. *, $P < 0.05$; **, $P < 0.01$; ***, $P < 0.001$; n.s., $P \geq 0.05$.

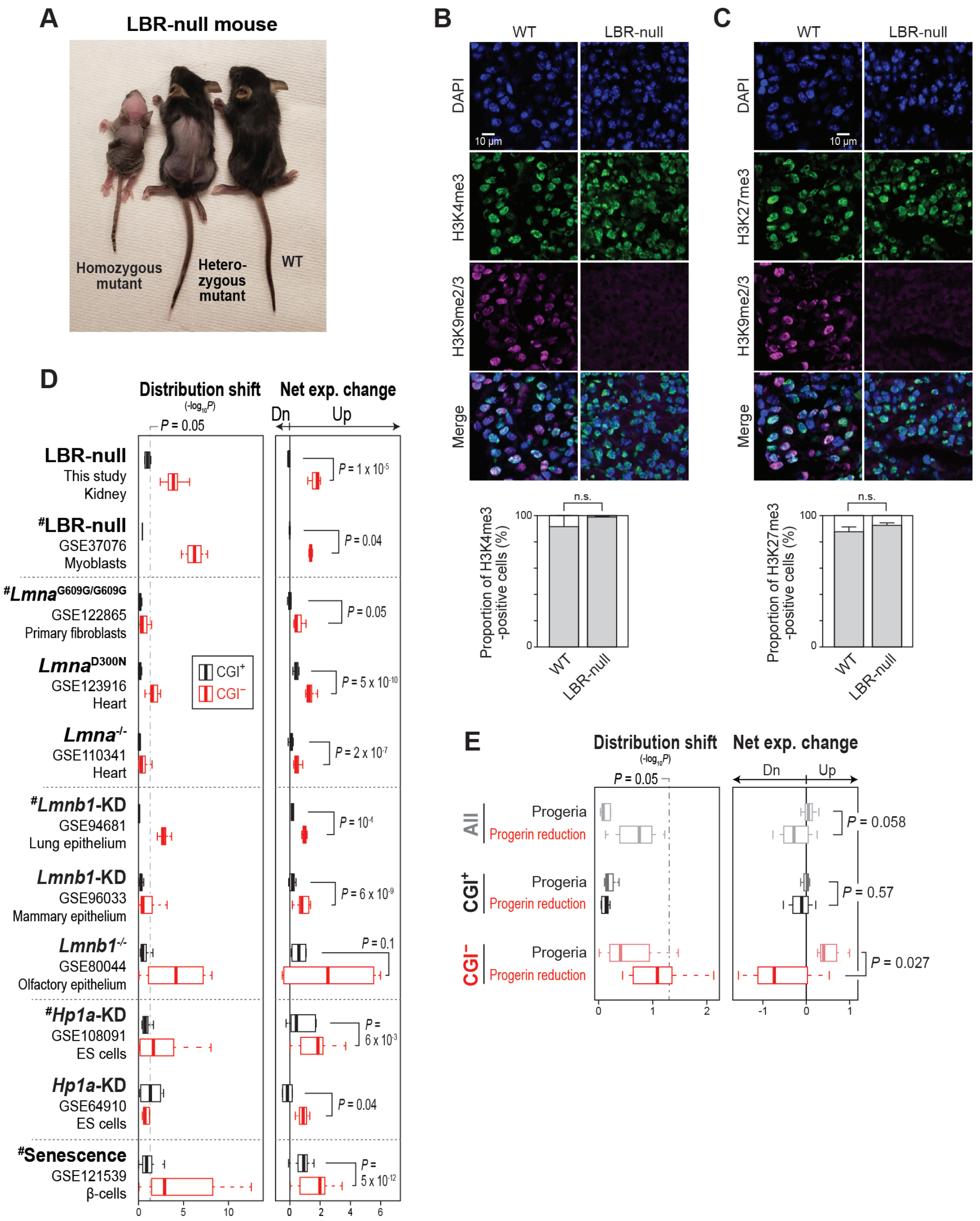

Fig. S2. Changes in gene expression upon disruption of chromatin architecture. (A) Homo- and heterozygous LBR-null mice and WT mouse. Note that LBR-null mouse displays sparse fur and smaller body size, both of which are commonly observed in mouse models of premature aging (*78*). **(B and C)** Immunofluorescence tissue staining combined with RNA-FISH in LBR-null mouse kidneys. H3K4me3 (**B**) or H3K27me3 (**C**) were detected along with H3K9me2/3 and CGI⁻ gene *Lcn2* misexpressed upon disruption of chromatin architecture. n.s., $P \geq 0.05$. **(D)** Distribution shifts (left) and net expression changes (right) upon chromatin architecture disorganization. #A representative dataset shown in Fig. 2. See table S4A for the full list of data. **(E)** *In vivo* effect of progerin reduction in progeria mice. Distribution shift and net expression change of progeria or progerin-reduced mice were calculated compared to control or progeria mice, respectively. RNA-seq data under accession GSE122865 were reanalyzed (table S4).

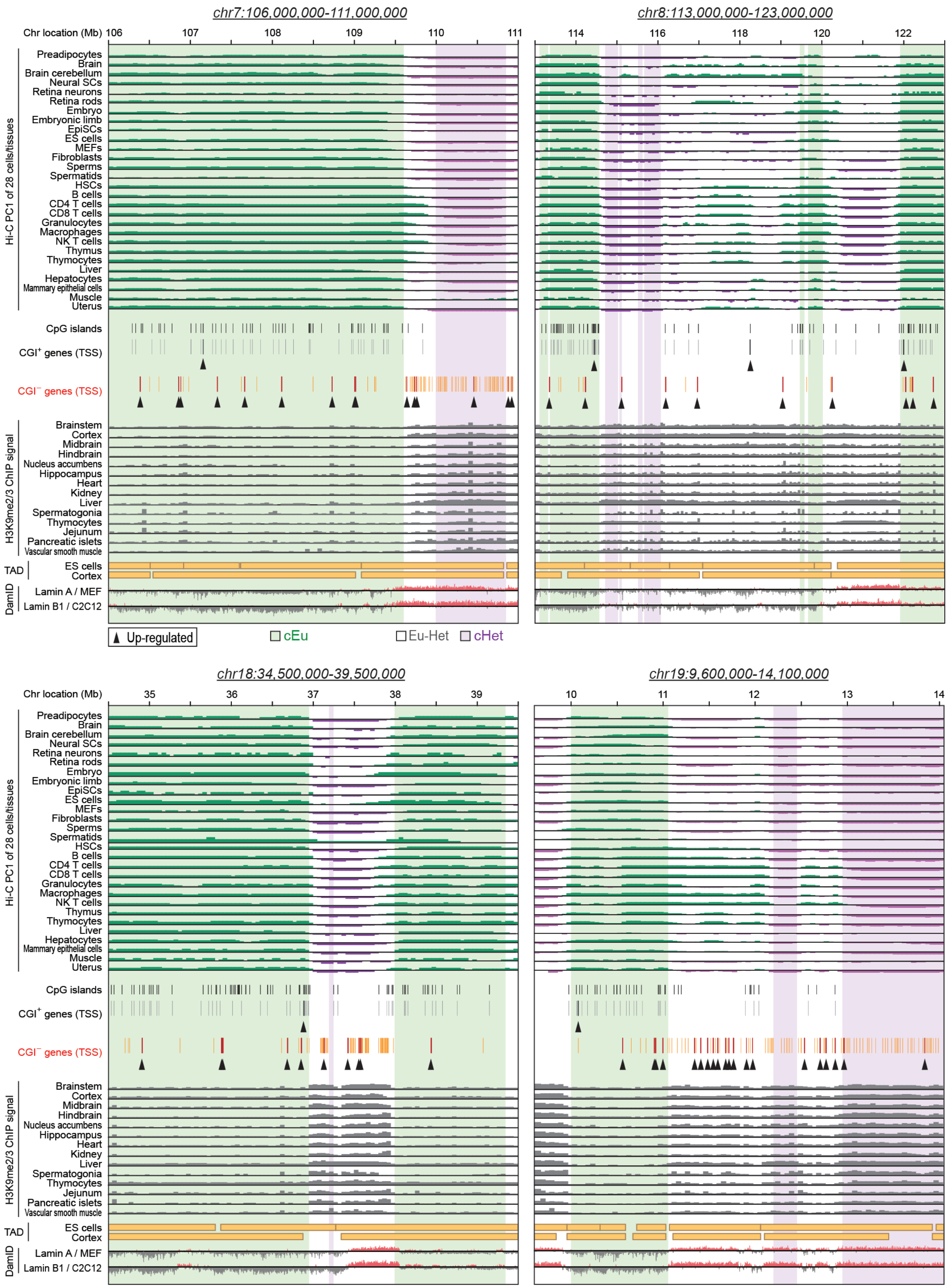
chr7:106,000,000-111,000,000
chr8:113,000,000-123,000,000
chr18:34,500,000-39,500,000
chr19:9,600,000-14,100,000
Chr location (Mb)
Hi-C PC1 of 28 cells/tissues
Preadipocytes
Brain
Brain cerebellum
Neural SCs
Retina neurons
Retina rods
Embryo
Embryonic limb
EpiSCs
ES cells
MEFs
Fibroblasts
Sperms
Spermatids
HSCs
B cells
CD4 T cells
CD8 T cells
Granulocytes
Macrophages
NK T cells
Thymus
Thymocytes
Liver
Hepatocytes
Mammary epithelial cells
Muscle
Uterus
CpG islands
CGI+ genes (TSS)
CGI− genes (TSS)
H3K9me2/3 ChIP signal
Brainstem
Cortex
Midbrain
Hindbrain
Nucleus accumbens
Hippocampus
Heart
Kidney
Liver
Spermatogonia
Thymocytes
Jejunum
Pancreatic islets
Vascular smooth muscle
TAD
ES cells
Cortex
DamID
Lamin A / MEF
Lamin B1 / C2C12
Up-regulated
cEu
Eu-Het
cHet

Fig. S3. Genomic landscapes of misexpressed CGI$^{+/-}$ genes across the mouse genome. Hi-C PC1 values of 28 cell/tissue types are shown in the upper section of each panel. H3K9me2/3 ChIP-seq signals, TADs (topologically associated domains), and DamID-seq signals are shown in the lower parts. CGIs and TSSs of CGI$^{+/-}$ genes are indicated by vertical bars.

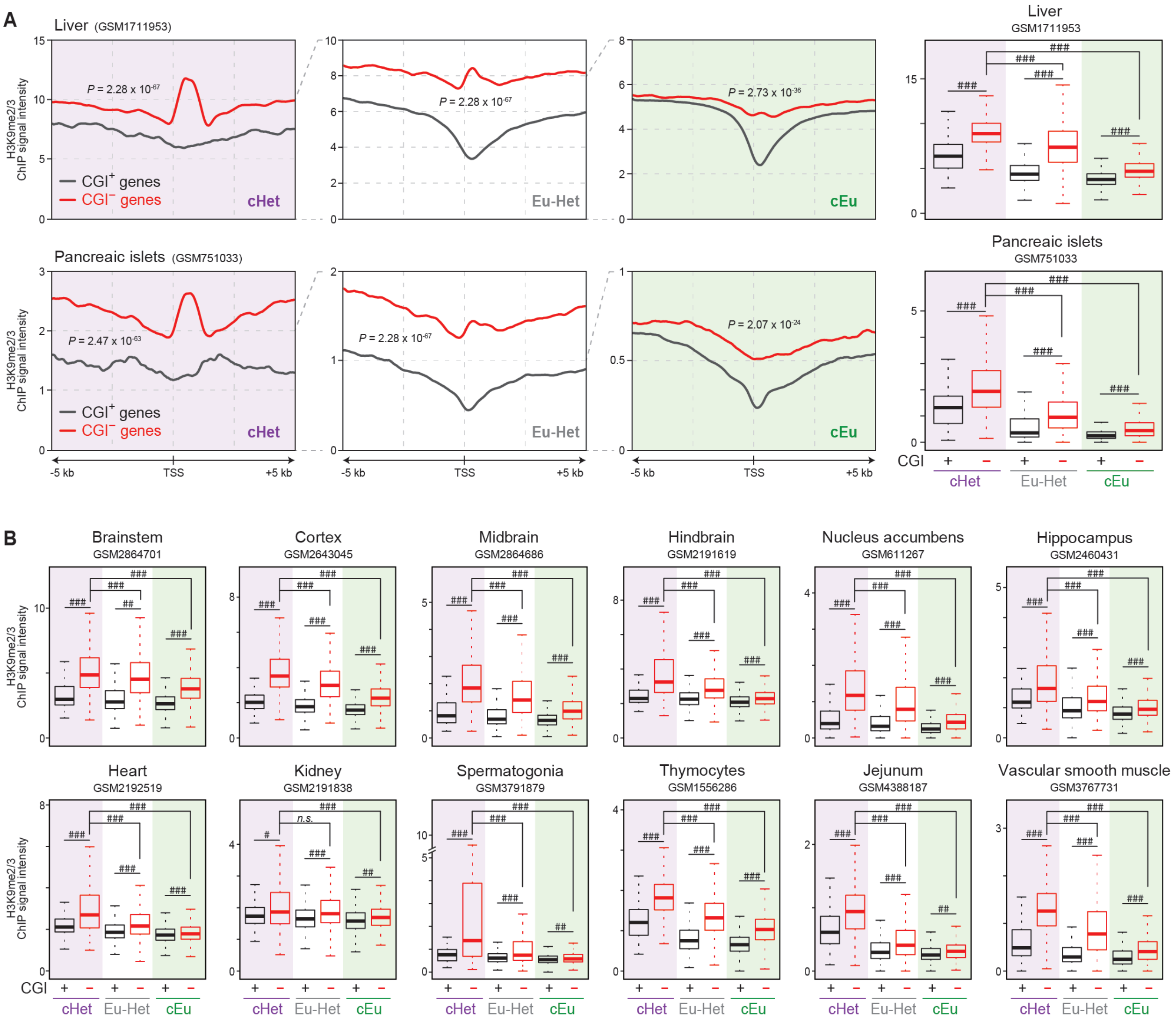

Fig. S4. Heterochromatin signals near transcription start sites (TSSs) in each chromatin domain. (A) H3K9me2/3 ChIP signal distributions in 10 kb windows surrounding TSSs in each chromatin domain of young liver and pancreatic islets (left) and their average signals within 2 kb-surrounding regions (right). **(B)** Average H3K9me2/3 ChIP signals within 2 kb surrounding regions of TSSs in various normal young adult mouse tissues. ChIP-seq data used in this analysis are listed in table S6.

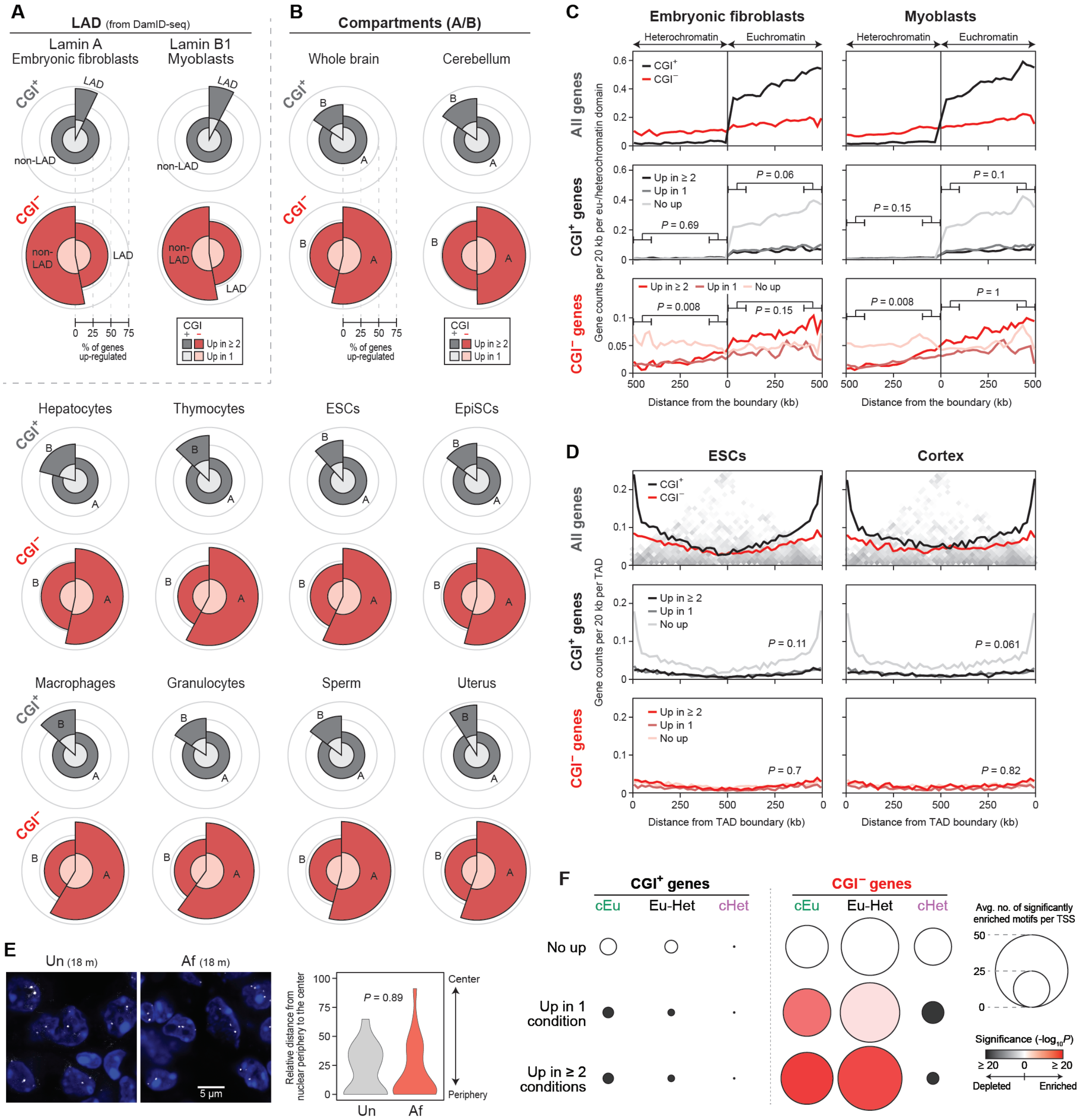

Fig. S5. Chromatin architecture context of CGI⁻ gene misexpression. (A and B) Proportion of genes within LADs/non-LADs (**A**) or compartment A/B (**B**) that are up-regulated upon disruption of chromatin architecture. LADs were defined by nuclear lamina (Lamin A or Lamin B1) DamID-seq. **(C)** Distance of genes from eu-/heterochromatin boundary. Note that up-regulated CGI^- genes within heterochromatic domains were more abundant at the boundary (i.e., near euchromatin) rather than at the center (bottom left of both data). Within euchromatic domains, proportions of up-regulated CGI^- genes near the boundary and center were not significantly different. Eu-/heterochromatin were determined using LAD maps. **(D)** Distance of genes from TAD boundary. Distribution of CGI^- genes are relatively even across TADs, while CGI^+ genes are significantly enriched near TAD boundaries. However, frequencies of misexpression were not significantly affected by TAD structures for either CGI^+ or CGI^- genes.

Publicly available TAD maps were used (*79*). *P*-values were calculated by comparing the proportion of genes in each 20 kb window. **(E)** Localization of multiple CGI^{-} genes misexpressed in affected DO mouse kidneys. 3D-FISH was performed to determine gene localization and distance from the periphery to the center. The relative nuclear location of these genes did not change significantly during aging. **(F)** Enrichment of transcription factor (TF)-binding motifs in $CGI^{+/-}$ genes. The number of known TF-binding motifs in a 500 bp-region surrounding the TSS of each gene are shown. Across the eight conditions shown in Fig. 2D, genes not up-regulated in any conditions ("No up"), up-regulated in only one condition ("Up in 1"), or recurrently up-regulated in two or more conditions ("Up in ≥ 2") are shown. Significance was calculated using hypergeometric distribution compared to the "No up". See also table S7 for the detail. $^{\#}$, $P < 0.05$; $^{\#\#}$, $P < 10^{-5}$; $^{\#\#\#}$, $P < 10^{-15}$; n.s., $P \geq 0.05$.

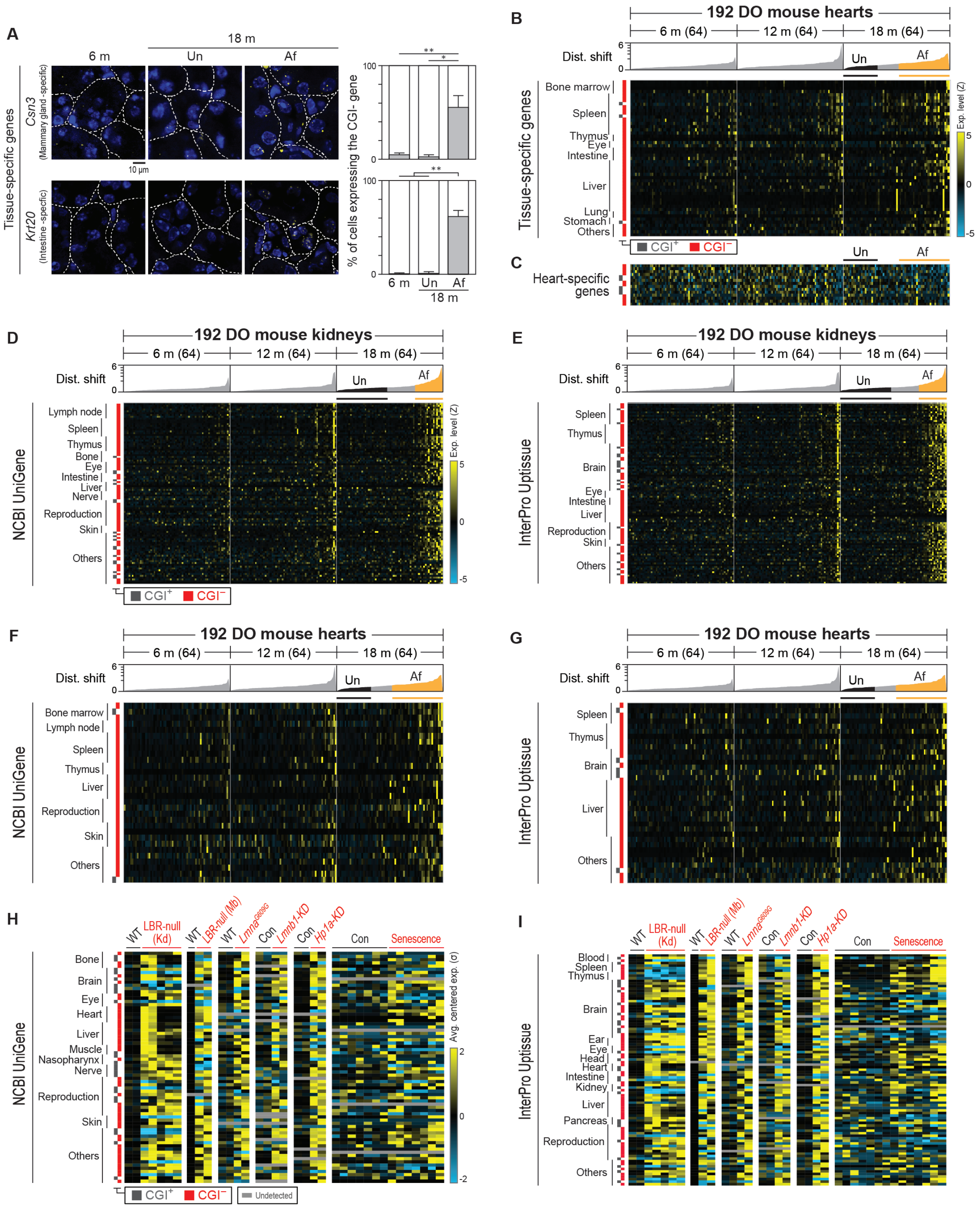

Fig. S6. Tissue-specific gene expression in kidneys and hearts of DO mice. (A) mRNA-FISH assays to detect misexpression of CGI⁻ tissue-specific genes. Note that tissue-specific genes that are not normally expressed in young kidneys were detected in DO mouse kidneys. White dotted lines indicate the boundaries of renal tubules. Error bars indicate standard deviation of three

replicates. *, $P < 0.05$; **, $P < 0.01$; ***, $P < 0.001$. **(B and C)** Expression of tissue-specific genes (**B**) and heart-specific genes (**C**) in DO mouse hearts. **(D and E)** Expression of tissue-specific genes defined by UniGene (**D**) and InterPro Uptissue (**E**) in DO mouse kidneys. **(F and G)** Expression of tissue-specific genes defined by UniGene (**F**) and InterPro Uptissue (**G**) in DO mouse hearts. **(H and I)** Expression of tissue-specific genes defined by UniGene (**H**) and InterPro Uptissue (**I**) upon chromatin architecture disruption. All genes shown in this figure are listed in table S9.

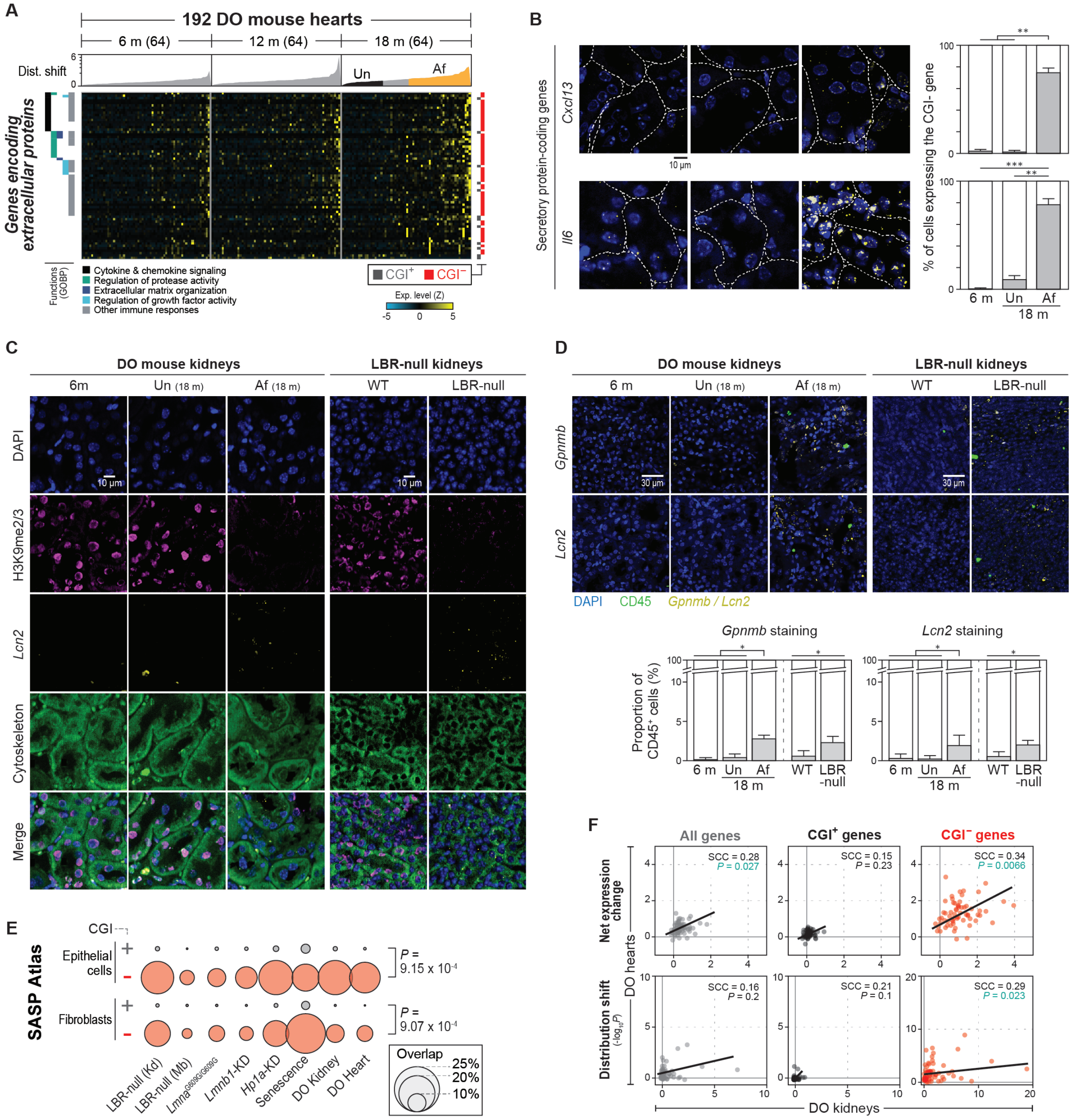

Fig. S7. CGI⁻ gene misexpression and uncontrolled secretory phenotypes during aging. (A) Expression of genes encoding extracellular proteins in DO mouse hearts. Shown are extracellular protein-coding genes which are significantly up-/down-regulated in affected hearts (versus unaffected, FDR < 0.05). For a full list of genes and their functions (GOBP, Gene Ontology: Biological Process), see table S11B. **(B)** mRNA-FISH assays to detect misexpression of secretory protein-coding CGI⁻ genes in DO mouse kidneys. White dotted lines indicate the boundaries of renal tubules. **(C and D)** Staining of cytoskeleton (**C**) or CD45⁺ leukocytes (**D**). Transcripts of CGI⁻ genes (*Gpnmb* or *Lcn2*) misexpressed in both DO and LBR-null kidneys were detected using mRNA-FISH. Note that the CGI⁻ gene *Lcn2* was misexpressed within affected and LBR-null renal tubular cells forming intact tubular structures. **(E)** Overlap among

previously defined SASP factors and CGI$^{+/-}$ genes misexpressed upon chromatin architecture disorganization and during aging. Human SASP factors obtained from the SASP Atlas (http://www.saspatlas.com/) were converted into mouse orthologs. **(F)** Spearman correlation coefficient (SCC) of net expression changes (top) and distribution shifts (bottom) between 18-month-old DO mouse kidneys and hearts. Linear regression analysis was performed using total least squares method. *, $P < 0.05$; **, $P < 0.01$; ***, $P < 0.001$. Error bars indicate standard deviation of three (**B**) or four (**C**) replicates.

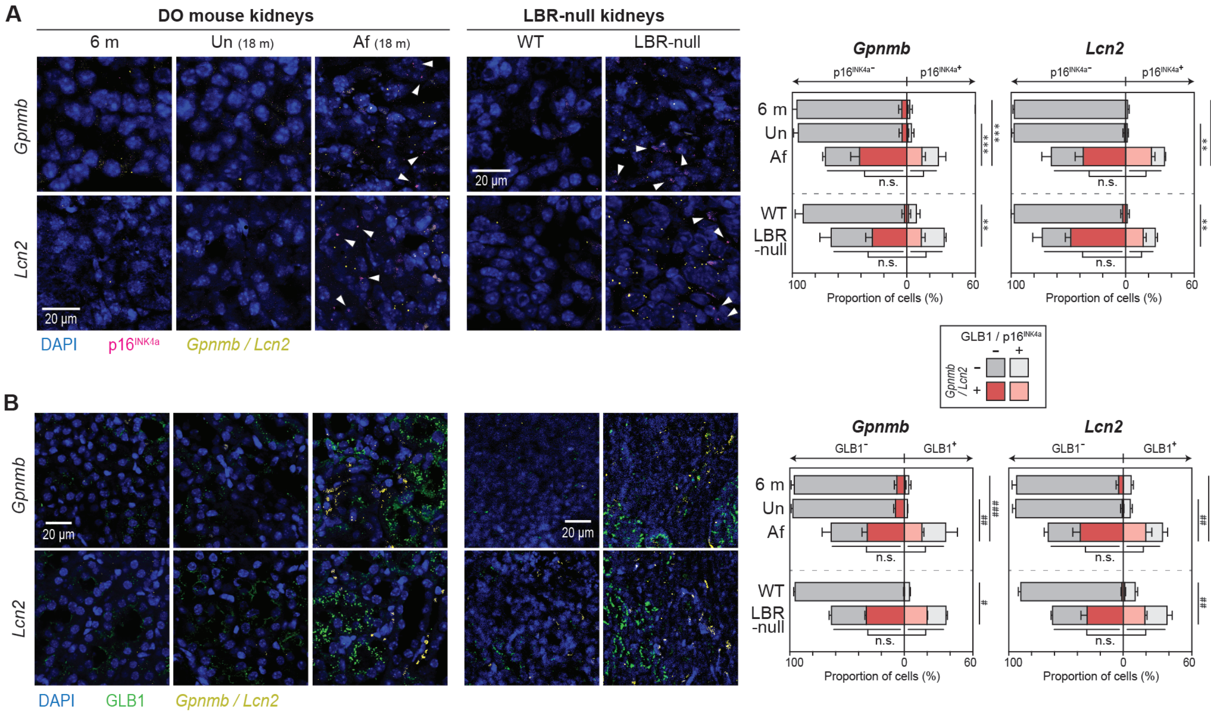

Fig. S8. CGI⁻ gene misexpression in senescent cells. (A and B) mRNA-FISH of p16^{INK4a} (**A**) and immunofluorescence staining of GLB1 (**B**). Transcripts of CGI⁻ genes (*Gpnmb* and *Lcn2*) misexpressed in both DO and LBR-null kidneys were detected using mRNA-FISH. White arrowheads indicate p16^{INK4a}-positive cells. Error bars indicate standard deviation of four replicates. *, $P < 0.05$; **, $P < 0.01$; ***, $P < 0.001$; #, $P < 10^{-5}$; ##, $P < 10^{-20}$; ###, $P < 10^{-30}$.

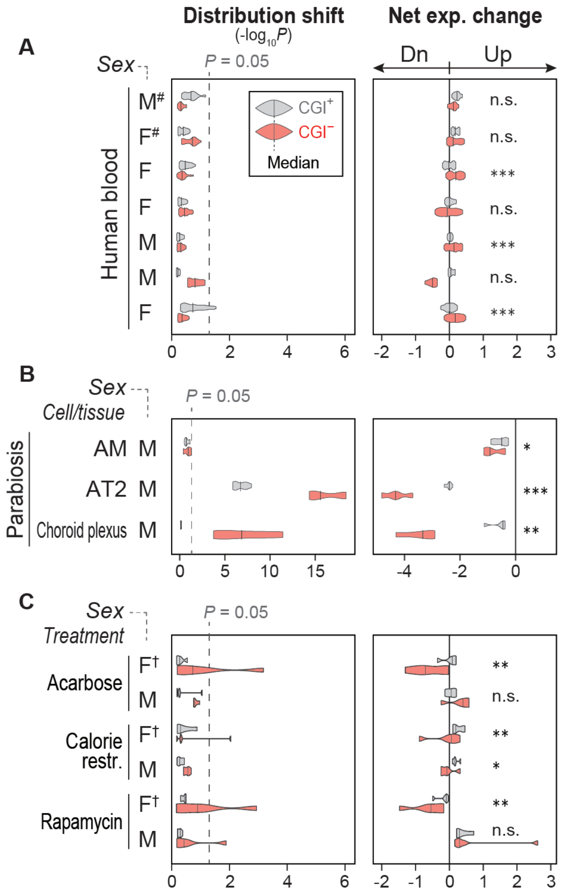

Fig. S9. Additional data in meta-analysis of published RNA-seq data (for Fig. 5). (A) Distribution shifts and net expression changes in human blood. #Also shown in Fig. 5A. **(B)** Effects of parabiosis on CGI⁻ gene misexpression. AM, alveolar macrophage; AT2, alveolar type 2 cell. **(C)** Sex differences across anti-aging interventions. †Also shown in Fig. 5C. Note that rapamycin and acarbose treatment were significant only in female mice, which is consistent with prior studies showing that mTOR inhibition was more effective in females (*80*). *, $P < 0.05$; **, $P < 0.01$; ***, $P < 0.001$; n.s., $P \geq 0.05$.

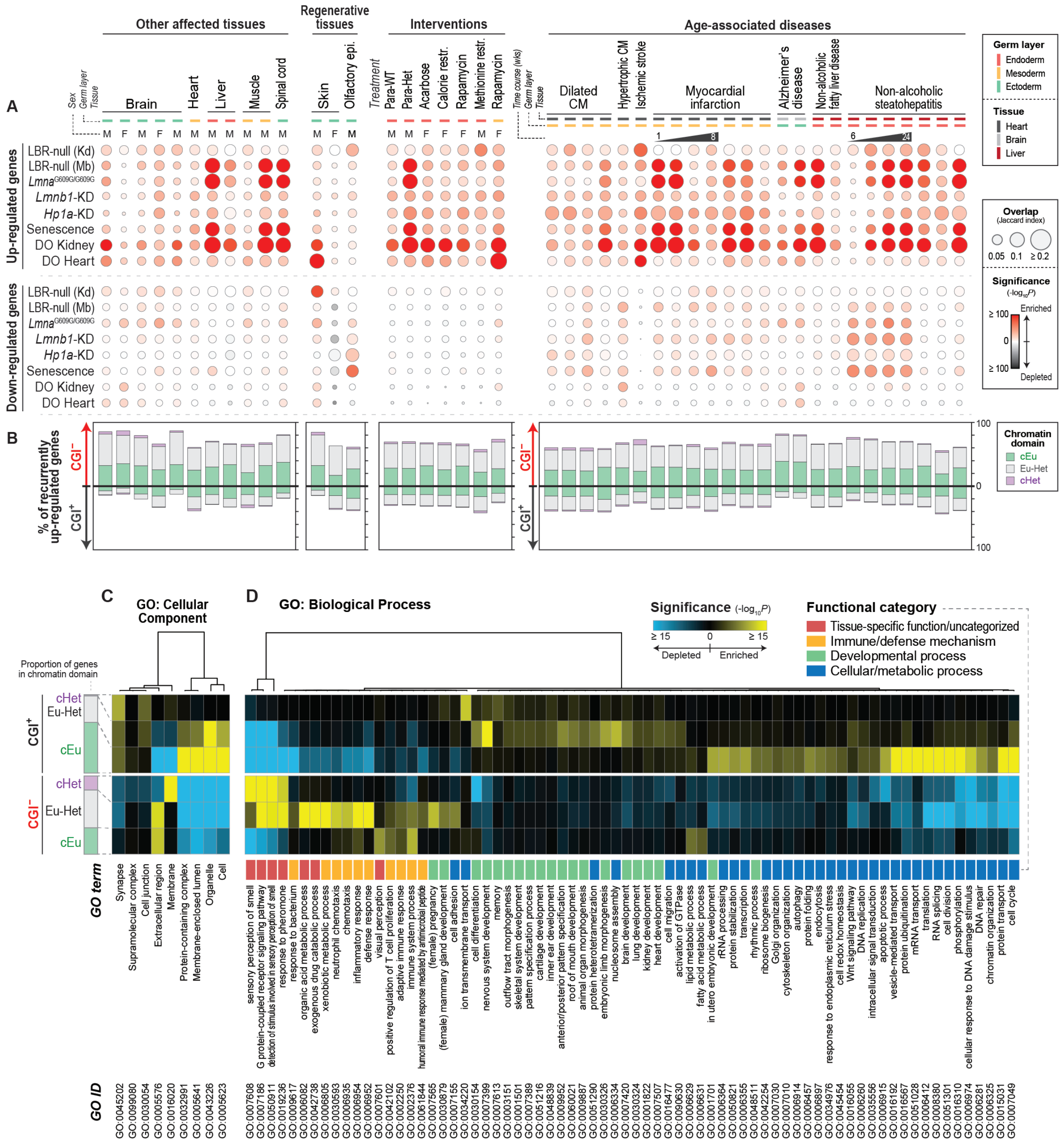

Fig. S10. Consensus of CGI⁻ gene misexpression upon chromatin architecture disruption and in aging/anti-aging intervention/age-associated disease contexts. (A) Overlap among differentially expressed genes. Significance was calculated by permutation tests (n = 1,000). **(B)** Percentages of repeatedly up-regulated genes. Overlaps between genes that are recurrently up-regulated upon nuclear architecture disruption and those up-regulated in each indicated dataset are shown. Note that up-/down-regulated genes resulting from anti-aging interventions were compared with down-/up-regulated genes upon chromatin architecture disruption, respectively, to demonstrate the counteraction of age-associated CGI⁻ gene misexpression. **(C and D)** Analysis of Gene Ontology (GO). GO: Cellular Component (GOCC; **C**) and GO: Biological

Process (GOBP; **D**) are shown. For GOCC, All depth-1 GOCC (the highest level in the GOCC hierarchy tree) are shown. For GOBP, GO terms with ≥ 50 annotated genes that are significantly enriched/depleted ($P < 10^{-6}$) in at least one chromatin domain are shown. $CGI^{+/-}$ genes were classified by their location in chromatin domains (cEu, Eu-Het, and cHet). Results were hierarchically clustered based on Euclidean distance to enhance visual clarity.

Captions for tables S1 to S12

Table S1. References used in fig. S1A. References having the experimental evidence of nuclear defects at protein level are given (PMIDs are shown).

Table S2. Mammalian CGI$^+$ and CGI$^–$ genes. (A) A list of mouse CGI$^{+/–}$ genes (mm9, NCBI build 37). **(B)** A list of human CGI$^{+/–}$ genes (hg19, NCBI build 37).

Table S3. Gene expression of affected and unaffected DO mice. (A) DO mouse kidneys. **(B)** DO mouse hearts. Differentially expressed genes: adjusted $P < 0.05$.

Table S4. RNA-seq used in Fig. 2C and fig. S2D. (A) A list of the datasets. **(B)** Differentially expressed genes upon chromatin architecture disruption.

Table S5. Hi-C data and chromatin domains used in this study. (A) A list of Hi-C datasets examined in this study. **(B)** Chromatin domains determined in this study (50 kb windows).

Table S6. ChIP-seq and DamID-seq data used in this study. (A) A list of ChIP-seq data used in this study. **(B)** A list of DamID-seq datasets examined in this study.

Table S7. Motif occurrence in TSS and its flanking region of CGI$^+$ and CGI$^–$ genes. (A) The number of known DNA motifs in 500 bp-region surrounding transcription start site of each gene. **(B)** Enrichment of motifs in the gene groups. FDR is shown. Genes were grouped by recurrency of up-regulation and assigned chromatin domains. Motifs enriched at least in one gene group (FDR < 0.05) were listed.

Table S8. Tissue-specific genes used in Fig. 3 and fig. S6, B and C. (A) RNA-seq datasets examined to define tissue-specific genes. **(B)** Tissue-specific genes defined by the meta-analysis in this study.

Table S9. Gene lists used in the heatmaps for tissue-specific gene analysis. (A) For Fig. 3A. **(B)** For fig. S6B. **(C)** For fig. S6C. **(D)** For fig. S6D. **(E)** For fig. S6E. **(F)** For fig. S6F. **(G)** For fig. S6G. **(H)** For Fig. 3C. **(I)** For fig. S6H. **(J)** For fig. S6I. Genes were sorted as shown in the figures. Genes labelled as “Others” in the figures are shown in blue fonts.

Table S10. Single-cell RNA-seq datasets used in Fig. 3E. *Author-provided age groups of each study were used for the analysis.

Table S11. Gene lists used in the heatmaps for secretory protein-coding gene analysis. (A) For Fig. 4B. **(B)** For fig. S7A. **(C)** For Fig. 4D. Genes were sorted as shown in the figure.

Table S12. RNA-seq datasets used in Fig. 5 and fig. S9. *A forward slash distinguishes groups (young/old or control/disease) if their ages are different.
